# Co-designing genomics research with a large group of donor-conceived siblings

**DOI:** 10.1186/s40900-021-00325-7

**Published:** 2021-12-16

**Authors:** Jack S. Nunn, Marilyn Crawshaw, Paul Lacaze

**Affiliations:** 1 School of Psychology and Public Health, La Trobe University, VIC Melbourne, Australia; 2 Science for All (Charity), VIC Melbourne, Australia; 3 Department of Social Policy and Social Work, University of York, York, UK; 4 Department of Epidemiology and Preventive Medicine, School of Public Health and Preventive Medicine, Monash University, Melbourne, Australia

## Abstract

**Background:**

Human genomics research is growing rapidly. More effective methods are required for co-design and involving people, especially those sub-populations which are inherently high interest to medical research and thus at greater risk of being exploited. This case study documents how we worked with a large group of donor-conceived siblings who share the same sperm donor father, to explore how they might want to engage with and influence any future genomic research.

**Method:**

A participatory action research process was used to explore the views of a group of 18 people who knew they are donor-conceived siblings. They are part of a larger group of up to 1000 people who share the same sperm donor father but the only ones in contact with each other; it is likely that many of the uncontacted siblings are unaware of their biological father, have been unable to trace others or have died. The discussion explored views about how the group would like to be involved in future research. Five members participated in co-design; 12 completed a pre-discussion online survey; and six participated in an online discussion forum and evaluation survey. The online discussion was led by one facilitator, supported by the study team.

**Results:**

Of the 18 siblings approached in 2018, 14 participated in the co-design stages or the surveys and online discussion. Co-design informed the research process. Participants reported enjoying the overall experience of the surveys and discussion forum, which were perceived as inclusive and flexible. Most participants’ views regarding the value of involvement in research changed during the process, and ‘widened’ about who should be involved. Participants were supportive of future research being done with the siblings group. All who completed the final survey requested to remain part of the co-design process. Other themes in the online discussion included concerns about conflicting interests and a desire for research participation to improve the situation for people affected by assisted conception. The process informed later discussions in the sibling group about participating in a self-managed biobank and informed decision making about participating in genomics research.

**Conclusion:**

Findings from this study help inform ways in which people from certain sub-populations can be involved in planning and defining their participation in genomic research, particularly those that are inherently high interest to medical research and thus at greater risk of exploitation. This process provides a replicable method of involving potential participants in co-designing genomics research using online discussions, with positive outcomes. Reporting this study using ‘Standardised data on initiatives (STARDIT)’ to report the process allows comparison with other studies.

**Supplementary Information:**

The online version contains supplementary material available at 10.1186/s40900-021-00325-7.

## Plain English summary

Human genomics research is growing rapidly. There is evidence that involving potential participants and the public in co-designing research can improve the quality, recruitment and acceptability of the research. However, more evidence about effective methods for involving people is required, especially those in sub-populations who are inherently high interest to researchers and thus with a higher risk of being exploited by medical researchers. In this study, we worked with a large group of donor-conceived siblings who were conceived from the same sperm donor. We sought their views regarding participation in possible future research. We co-designed a way of involving them in discussion about their own “terms of engagement” with research. Online discussions gave group members an opportunity to share their views, and take initial steps towards developing their own research governance model. We used the ‘Standardised Data on Initiatives (STARDIT)’ Alpha Version to report involvement, allowing findings to be compared with other studies. Group members who participated reported enjoying the experience and identified some advantages to online discussions over other methods, including time to reflect on answers and learn collectively. Most participants’ preferences about who should be involved in research design ‘widened’ to include more people. Participants’ learning from the process also informed subsequent discussions in the sibling group about participation in research, including about how to make informed decisions about participating. Involving people in this way has the potential for a ‘transformative learning’ impact, where people’s perspectives and attitudes change as a result of being involved. This is particularly important for people in populations at greater risk of being exploited including under-represented sub-populations who are of high interest to genomic researchers.

**Supplementary Information:**

The online version contains supplementary material available at 10.1186/s40900-021-00325-7.

## Introduction

Human genomics research involves defining sub-populations, measuring DNA changes within them and linking them to traits or outcomes in order to understand how DNA variation can contribute to human health and disease. The more genetically similar people are, the more likely it is that they will share the same DNA variations that contribute to a given trait such as wellness or disease. Therefore, historically human genomic research has focused on restricted populations who share common biological ancestry, including large families or founder populations, where less genetic variation has led to more clear links between DNA and disease [1].

Since the advent of affordable online ancestry DNA testing, increasing numbers of people are taking DNA tests to understand genetic ancestry (genealogy). However this DNA testing can sometimes result in returning unexpected information, including the revelation of being sperm donor-conceived [2]. These genetic results can also immediately link people to a broader group of biologically-related people who also share the same biological father [2].

Owing to the increasingly affordability of direct-to-consumer DNA testing [2], a growing number of large groups of donor-conceived siblings are now being discovered, some with over 100 half-siblings [3, 4]. In some cases, people from such sibling groups were conceived before regulation or clear legal oversight of assisted conception services. For example, in the UK, a government register of donors was proposed as early as 1949 [5], but such a register was not established until 1991 [6]. By 1958 the total number of donor conceived people in the UK was estimated to be 7,500, and 100,000 in the United States [5]. Some countries, including developed economies such as Japan, still have no clear legal regulation or oversight of human sperm and egg donation [5, 7, 8].

A historical and contemporary lack of regulation means that there may be many undiscovered large sub-populations of individuals who share a common sperm donor. Such sub-populations may provide new insights into medical, genetic, sociological and psychological studies, and therefore are of inherently high interest to research, including genomics research. However, such interest also makes them at greater risk of exploitation [9–11]. There are prior examples of other groups with shared biological ancestry having research conducted “on them” rather than in partnership “with them” [12–14]. The preferences of people within these sub-populations about how they would like to work with researchers conducting research with them, and how they would like to be involved are yet to be established [12].

Here, we involved people who know themselves to be members of a donor-conceived sibling group in co-designing this research. This case study documents a participatory action research process with members of one of the largest biologically-related (donor-conceived) family groups ever documented (hereafter ‘the sibling group’) [3, 5, 15–17].

Members of the sibling group are all offspring of one sperm donor, the scientist Dr Bertold Wiesner, a consulting biologist at the Royal Northern Hospital in the 1940s [18]. He may have fathered up to 1,000 offspring between 1942 (or earlier) and 1967 [5, 16, 17] through the medical practice of his wife Dr Mary Barton, despite a 1945 British Medical Journal paper where Barton and Wiesner stated they set an ‘arbitrary limit of 100 children for each donor’ [19]. This study represents the first of its kind done with this group of siblings. The 18 siblings known to each other at the time of this research project are the living members of the cohort who are both aware that they are part of a larger group of up to 1000 people who share the same biological father, and have chosen to make ongoing contact with each other (hereafter ‘known siblings’).

A formal database of people descended from Wiesner is not known to exist. It is therefore possible that some descendants have lived their whole lives and then died, without knowing their biological father was Wiesner. The purpose of this research was to involve known siblings in online discussions about how they would like to be involved in future research, to better understand preferences of the group members in order to inform any future genomics research. More information about this sibling group and relevant contextual and historical information can be found in Additional file 1.

We have also reported the participatory action research process and outcomes using the novel tool ‘Standardised Data on Initiatives (STARDIT)’, which is an open access data-sharing system to standardise the way that information about initiatives is reported [20–22]. More detailed information about STARDIT can be found in a separate article about the Beta version [23].

## Methods

### Study design

A participatory action research method was chosen for the study, with co-design and reporting guided by a number of frameworks [24–26]. ‘Participatory action research’ is an umbrella term which describes a number of related approaches, including forms of action research which embrace a participatory philosophy and include ‘co-design’ and ‘co-production’ of research [27, 28]. It is a process where researchers, relevant stakeholders and sometimes the public “work together, sharing power and responsibility from the start to the end of the project” [29], including knowledge generation and translation [29]. The term ‘stakeholder’ means anyone who has a ‘stake’ in the research, in particular those with important knowledge, experiences, expertise or views that should be taken into account [30, 31].

After one of the investigators (JN) discovered his biological grandfather was Bertold Wiesner, the study team invited a researcher who had previously worked with members of the sibling group (MC) to join the study team. The biological relationship of the investigator JN was then used as a starting point by the study team to work closely with both potential participants and experts in human research ethics from the La Trobe University to ensure the method was acceptable and that no one (including the study team) would be exposed to avoidable risk. Guidance on ethical involvement of potential participants in research design remains unclear [24], in particular when it involves individuals conducting research with biological relatives [32]. Accordingly it was decided by the study team to commence co-design only after ethics approval was obtained. Feedback from the co-design process was later integrated into the study design through multiple subsequent modifications to the initial research plan, with each modification being reviewed and approved by the Human Ethics Committee.

**Fig. 1:**
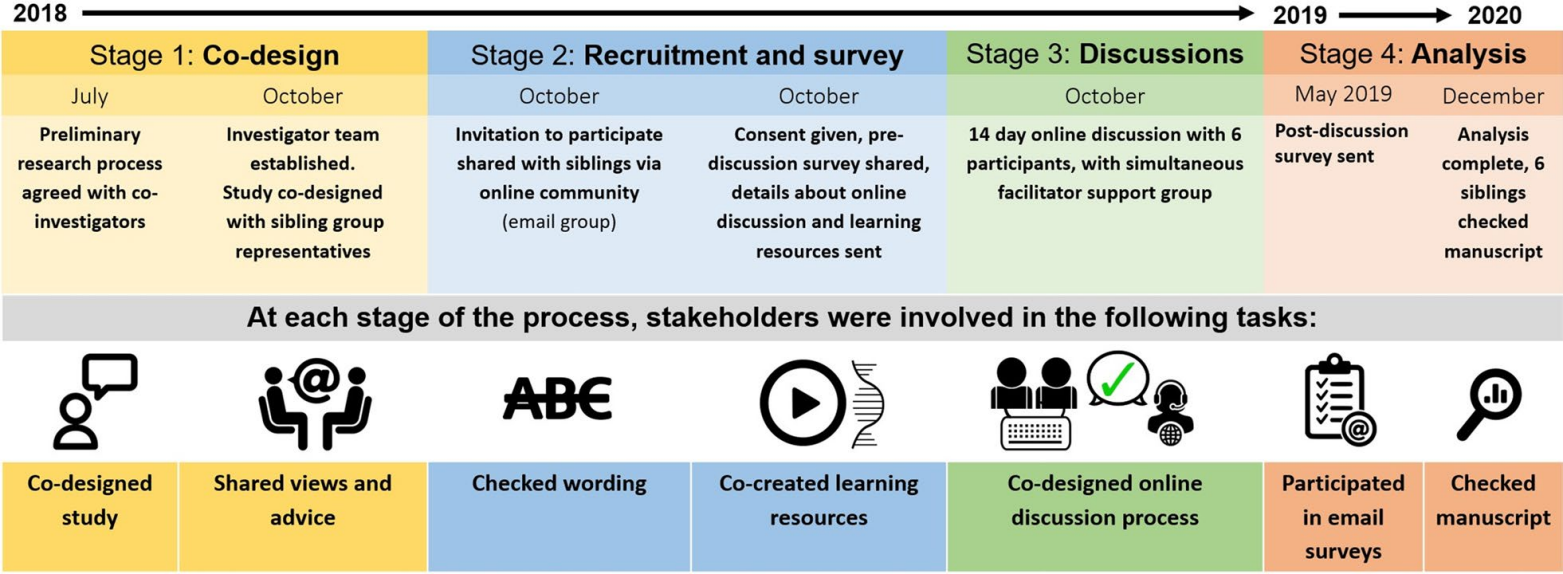
Stages of research

The study had four stages: 1. Co-design; 2. Recruitment and pre-survey completion; 3. Online discussion and post-survey and 4. Analysis, with stakeholders involved in tasks at each stage (Fig. 1).

However, reporting of such involvement is often inconsistent, of variable quality [12, 33], or the reporting frameworks themselves can have limitations [34]. STARDIT was used to report involvement, as it overcomes some of the identified limitations of other reporting tools by allowing people to self-identify with multiple labels such as ‘researcher’ and ‘patient', and by transparently allowing multiple stakeholders to be involved in reporting which stakeholders have completed which tasks [21]. It has been proposed as a way of describing the ‘who’, ‘how’ and ‘what’ of research, and as a way to report two-way learning and ‘transformational learning’ amongst other impacts [35, 36].

**Fig. 2:**
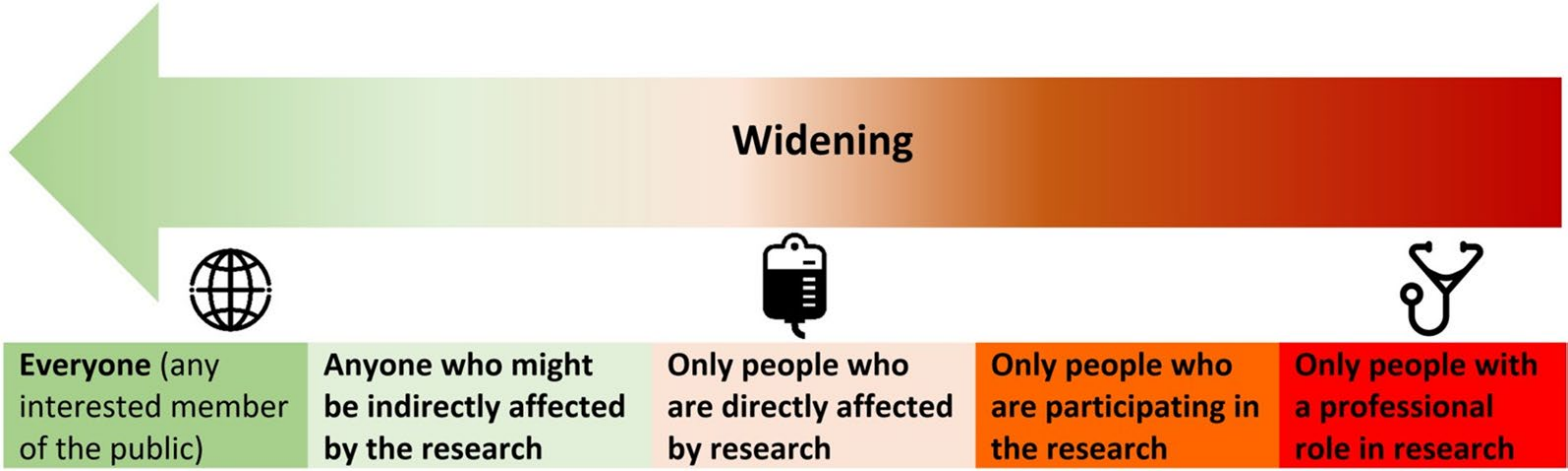
Who should be involved in research

Some elements of this study were co-designed in parallel with another similar study in order to facilitate comparison in data [36]. Accordingly, some aspects were inflexible and thus more accurately described as ‘co-refined’ rather than ‘co-designed’. For example, questions in the pre- and post-surveys relating to ‘who should be involved in research’ and were designed to explore any changes in views on which stakeholders should be involved in which tasks during research, and were worded identically in each study in order to allow comparison with standardised data, using the STARDIT Preference Mapping tool [20, 23, 36]. Participants could choose from the categories outlined in Fig. 2, with a change in direction towards more people being involved labelled as ‘widening’, the inverse as ‘narrowing’. The facilitator (MC) was also asked to complete a survey, which was informed by the Public Involvement Impact Assessment Framework Guidance (PiiAF) [25] and the questions were informed by the GRIPP2 reporting checklist [26]. The survey was identical to a survey used in other studies to facilitate data comparison [36, 37]. All survey questions can be found in full in ‘Additional file 1—Data and analysis’.).

**Fig. 3:**
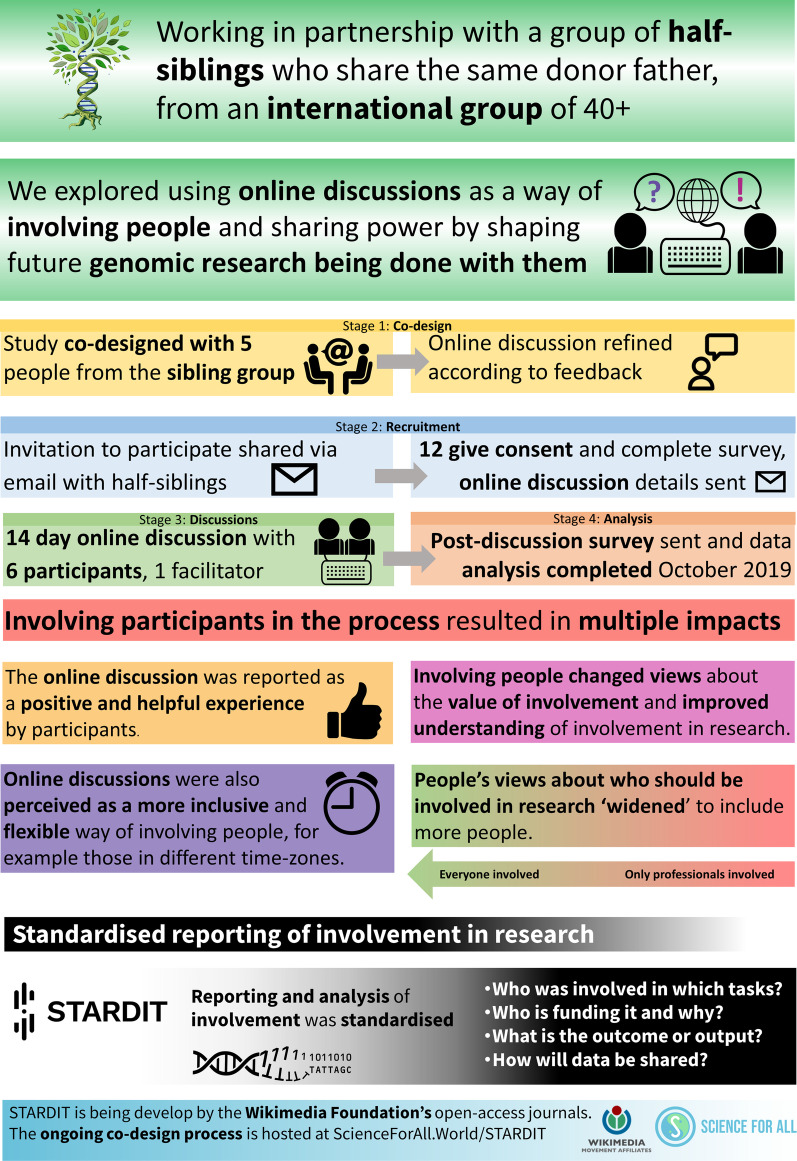
Summary of the research process

Similarly questions about demographic information for the two studies were informed by the Genioz study to allow comparison with a wider dataset [36, 38]. The choice of the software used in the online discussion was also fixed owing to ethical constraints, including physical data location and ownership by participants (Fig. 3).

Online methods of discussion are appropriate where a group of people is geographically dispersed and face-to-face discussion is impractical [39]. We therefore selected an online text-based discussion format, where people could log on at any time and contribute (asynchronous), including replying to other people’s comments (threaded discussion). This platform allowed flexibility compared to ‘real time’ (synchronous) discussion, especially regarding participants contributing from different time-zones. An open-source software web application called ‘Loomio’ [40] was installed on virtual machines hosted by an Australian Government initiative called ‘Nectar Cloud’ [41]. Participants could securely log-in from anywhere in world and participants’ data was stored securely on servers physically located inside Australia.

The online discussions were facilitated for 14 days and led by one facilitator (MC). The facilitator was trained in advance to use the online discussion platform, given learning resources, invited to be part of a community of practice of other facilitators running similar discussions [36], and given support via telecommunications (by JN) to discuss any emerging issues. At the start of the online discussion, the facilitator asked participants to consider agreeing on boundaries relating to acceptable conduct, and invited members of the discussion to co-create a group agreement on conduct. The facilitator judged when to introduce new topics (depending on the engagement with each topic) with the recommend schedule below in Table 1 used as a template. The facilitated discussion period also included the provision of learning materials (short videos, infographics and information about research terms). These learning resources are included in ‘Additional file 1—Data and analysis’.

**Table 1:** Discussion overview

Question	Suggested staging point
Agreeing boundaries	Day one
What do you understand by the word ‘research’?	Day one
What do you understand by the phrase ‘genomic research’?	Day one
Why do we do research?	Day two
Which aspects of any future research genomic research should be influenced by the which groups of people?	Day four
What methods do you think could be used to involve those people in future genomics research?	Day six
Do you have any ideas, thoughts or reflections that have not been shared yet?	Day seven
Discussion closed	Day 14

The learning resources provided during data collection drew on the outcomes of an analysis of educational materials by the Australian Genomics Health Alliance [42]. Resources were selected by the investigator team in partnership with the Australian Genomics Health Alliance and refined following feedback from the volunteers. Detailed information about which learning resources were shared at which stage of the study can be found in Additional file 1.

### Data analysis

We collected and analysed both qualitative and quantitative data during the participatory action research process. Data sources included pre- and post-surveys; online text-based discussions; meeting notes; emails between the study team members; surveys of the study team; comments shared by two of the study team (JN, MC) during the online text-based discussion; and reflexive research diary entries of one member of study team (JN) (Table 2). The data collection and analysis was informed by a number of frameworks and approaches for conducting and reporting qualitative research [43, 44]; and conducting [45–58] and reporting case studies [20, 59]. Participatory research processes, including stakeholder involvement, were informed by frameworks and best-practice for conducting [31, 60, 61], reporting [20, 21, 62], and assessing involvement in research [25].

**Table 2:** Summary of data analysis

Data source description	Analysis method
**Meetings**—including meeting notes and relevant documents	**Qualitative** (thematic analysis)
**Online survey**—text data from open and closed questions	**Qualitative** (thematic analysis, STARDIT-PM) **Quantitative** (number of responses)
**Online discussion**—text data from a facilitated and moderated online discussion of both participants and a separate one for the study team	**Qualitative** (thematic analysis, STARDIT-PM) **Quantitative** (number of responses)
**Study team surveys**—responses by email	**Qualitative** (thematic analysis, STARDIT-PM)
**Other data**—reflexive research diaries,	**Qualitative** (thematic analysis)

Coding and thematic analysis of qualitative data was carried out by two authors (JN, MC) independently; the analysis was then checked (triangulated) for validity by a third author (PL), and participants were invited to review the analysis (‘member checked’), which is best practice for enhancing validity in qualitative methods [55]. In addition to quantitative analysis, each source was analysed using the method of thematic analysis, which involved stages including data mapping and familiarisation, transcription, coding, searching for themes, reviewing themes with study team members, labelling and summarising themes, and reporting the findings [55]. Participants were invited to review the analysis before publication in order to check whether they felt it reflected the research process accurately. They were also invited to share any further data via the STARDIT report [22].

For the online discussion, we also used case study methodology to record and describe our experience of involving participants. This process is presented as an ‘instrumental’ case study, where the purpose is to understand the particular case, and attempt to provide data that could produce useful generalisations by using inferences from the data [51]. The co-design of the case study was informed by best practices for enhancing validity and rigour in the case study methodology [45, 47–50, 54–57, 63, 64]. In order to aid analysis and comparison with other case studies, we used ‘Standardised Data on Initiatives’ (STARDIT) Alpha Version to consistently map preferences for involvement and report ways people were involved in the participatory action research process [20]. Both the STARDIT report and the preference mapping can be found in the additional files. Further information about the case study method, data and both qualitative and quantitative analysis is provided in ‘Additional file 1—Data and analysis’.

### Participants and recruitment

The selection of the particular shared ancestry population for this case study was informed by a number of factors which were appraised by the study team, including pragmatic considerations coupled with extensive consultation with professional ethics advisors and other experts [53].

In October 2018 one member of the sibling group forwarded an email invitation to the other 17 known members to join the study. The invitation to participate contained a link to the participant information document; a plain English summary about the study; some learning resources about genomics research; and an informed consent form. We offered the choice of anonymity (through using pseudonyms or temporary email accounts) in survey completion and the online discussions to allow people to participate without disclosing sensitive or personal information.

Those who gave consent were invited to complete the online pre-discussion survey and were then contacted by email directly by a member of the study team (MC), who shared information about joining the online discussion alongside relevant learning resources. This included a short 60s online video about the study, giving information about the context and purpose, and a one-page infographic summary of a scoping review about involvement in genomics research [12].

## Results

### Stage 1: Co-design

**Fig. 4:**
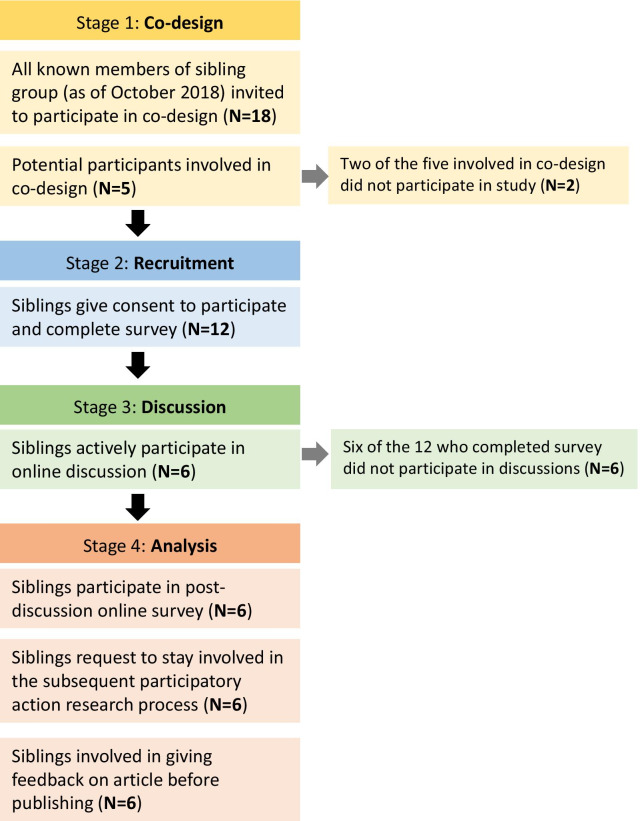
Sibling group involvement and participation

Concepts such as ‘co-design’ and ‘co-creation’ are used here to describe involving people in the respective tasks such as helping design the research project, or providing input during the creation of a learning resource. These concepts can be considered as part of participatory action research [27]. Five members of the sibling group gave feedback during the co-design process (Stage 1), three of whom went on to participate in the study. Their input during the planning and co-design stage had clear positive impacts, particularly in improving educational resources and ensuring the pre-discussion survey used appropriate and acceptable wording to describe the sibling group, the members and the sperm-donor. During the co-design process it was agreed that the initial study should only involve the siblings, excluding any of their offspring. It was agreed during the co-design process that direct communication with the sibling group would be conducted by a biologically unrelated member of the study team (MC) once the study commenced, owing to the ethically novel situation of one of the investigators (JN). Figure 4 summarises how many people participated in the stages of the study. Of the 18 known living siblings in 2018, 14 participated in the co-design stages or the study.

### Stage 2: Recruitment and surveys

Of the 18 members of the donor sibling group, 12 gave consent to participate and completed the pre-discussion survey of whom six participated in the online discussion. All six completed the follow-up survey in May 2019 and shared consistent identifying data at all stages, including the initial survey, the online discussion and the follow-up survey, allowing a comparison of results throughout the process.

Of the 12, seven were female and five were male. All stated that they were comfortable describing other descendants of their biological father as ‘half-siblings’. Self-reported educational attainment was mostly ‘degree (bachelors), diploma or post-graduate’, with one participant having professional experience in medicine and genomics. All were aged between 50 and 74, with most living in the UK. Most agreed with the statement that they have a shared interest in discussing future research which might affect them, including genomic research. More information about participant demographics can be found in Additional file 1.

From the six participants who completed both the pre-discussion and follow-up surveys, a total of 54 responses were given. Of these, 35% showed a change towards ‘widening’ involvement (N = 19/54), 8% ‘narrowed’ (N = 8/54) and half stayed the same (N = 27/54). More detailed data is provided in Additional file 1.

### Stage 3: Online discussions

Six people participated in the online discussion in October 2018. Only one chose to use a pseudonym in the online discussion, so the study team could identify them. Some of those who gave consent to participate did not make comments but logged-in multiple times and read comments. Table 3 shows how many comments were made by each participant in the online discussion. The themes where most participants shared views are summarised in Table 4. The most discussed themes included the unique nature of the sibling group, that anyone should be involved in research, questioning who decides ethical oversight in research, questioning research for profit and sharing concerns about genomics research being used for political purposes.

**Table 3:** Number of comments in online discussion

Participant ID	Number of comments
P2	12
P4	14
P5	42
P6	15
P7	9
P9	13
Facilitator (MC)	65

**Table 4:** Quantitative summary of themes

Theme	Number of participants
Anyone should be involved in research	6
Research with sibling group is unique and complex but important	6
Those affected by research should be involved	6
Research for profit and ‘bullying’ by ‘big pharma’	6
Who decides who decides what is ethical?	5
Concerns about genomics research being used for political purposes	5
Finding out they are part of sibling group has been a positive experience	4
View on topics for research	4
Participants reported changed views and perspectives as a result of participating	4
Desire to improve situation for people affected by assisted conception	3
Interested in learning what other siblings think and discuss issues	3
Concerns about control of knowledge and data	3
Questioning which groups should have ‘equal influence’?	3
Questioning eugenic attitudes to genomic variations	3
Views on participation in genomics research	3
Participants learned about genomics	3
Motivation for participation to help researchers and sibling group	2
Uncertainty about what they can offer but happy to help	2
What is the purpose of research?	2
Experts should be involved (over seen by ethics boards)	2
Developments in genomics have significant implications for society	2

### Stage 4: Analysis

Participant feedback in the post-discussion survey was positive, including that the experience was ‘interesting’, and that they ‘enjoyed thinking about the questions posed and reading the responses of others’ and the ‘perceptive comments’ of the facilitator [P7] [P4]. Two reported experiencing some usability issues with the online platform. All who completed the survey opted to stay involved in the next stages of the participatory action research process. A summary of pre and post survey responses can be found in Table 5.

**Table 5:** Summary of pre and post survey responses

Question	Results
**What made you decide to respond to our invitation to participate in this project?** 12 participants responded (pre-discussion)	Participants wanted an ‘opportunity to be involved in research’ [P3], wanted to ‘learn more’ [P7] and regarded involvement as a ‘civic duty’ [P10]. Others stated this study may be useful to ‘future donor offspring’ [P11] and one participant stated ‘curiosity’ was a reason for participation. Four participants noted a familial connection to a member of the study team (JN)
**What do you hope to get out of participating in this discussion? Do you have any specific expectations?** 8 participants responded (pre-discussion)	Participants were ‘interested in hearing what their half siblings think’ [P4] and wanted an ‘opportunity to discuss’ and ‘think through the issues involved’ [P4] [P6]. One participant said they wanted ‘the satisfaction of knowing that I may have contributed’ to the study [P10]. Another wanted ‘to be useful to the researchers’ [P11]. One participant noted an expectation of anonymity while participating
**Do you have any ideas about how the different people could influence future research?** 7 participants responded pre-discussion and 3 post-discussion	Five participants said ‘everyone should be involved in research’, with one adding ‘not just scientists and researchers’ [P5-pre]. One participant said ‘anyone with an opinion’ should be involved [P3-pre]. Another stated ‘researchers and those who are affected by what is being researched’ [P4-pre]. One participant stated ‘People who know their subject but do not have hidden motives or agendas’ should be involved [P2-pre]. One participant said the answer depended on ‘what kind of research it is’ [P7-pre] and one mentioned ‘ongoing discussions’ using online tools [P5-post]
**Is there anything in particular you liked or thought was helpful about how the discussion was conducted?** 5 participants responded (post-discussion)	One participant stated they ‘liked and appreciated the opportunity to participate’ [P6]. One participant stated the process ‘seemed to work well’ [P7]. One participant added ‘I think it is commendable that there is a concern about participatory research’ [P9]
**Is there anything you didn’t like, thought was unhelpful or could have been improved about how the discussion was conducted?** 5 participants responded (post-discussion)	Two participants reported finding the platform ‘complicated’ and problematic [P5]. Two participants stated they would have liked ‘more time’ for the process [P4]
**Did you have any expectations from participating in this research that were met or not met?** 6 participants responded (post-discussion)	Four participants stated their expectations were met. One responded that they ‘found some of the questions very complex and had difficulty answering them’ [P5]

### Outcomes from the process

There were 8 outcomes reported from this process, which are summarised in Table 6 with additional information available in the accompanying STARDIT report (Additional file 2).

**Table 6:** Summary of outcomes from the process

Outcome	Summary and learning point
1. Improved understanding of genomics informed participation in future research	Participants reported their understanding about genomics research increased as a result of participating in the study. Learning from this process informed subsequent discussions in the sibling group about participation in research, including a proposed self-managed biobank
2: Learning resources useful	Participants reported finding the information resources and videos useful. **Learning point:** Creating learning resources in multiple formats (hyper-text, infographic summaries, videos with subtitles animations) will ensure that information is more useful for people with neuro-diverse learning needs
3: Changed views and perspectives as a result of participation	Participants reported their views and perspectives changed as a result of participating. **Learning point:** Online discussion facilitated collaborative learning and the changed views of participants can be viewed as an impact of ‘transformative learning’ [39, 69, 70]
4: Participants asked to stay involved in the research	All participants who completed the follow-up survey opted to stay involved in the research process. **Learning point:** Participants demonstrated ‘critical reflexivity’, a stage of participatory action research which asks people involved to reflect on their views and the causes of problems and to be involved in exploring any solutions and the actions that people can take to bring about improvements [69, 71, 72]
5: Participants enjoyed the online discussions	Participants stated the experience of participating was ‘interesting’ and they ‘enjoyed’ it [P7] [P4], despite some usability barriers. **Learning point:** Some participants stated they preferred online discussions over face to face discussion or interviews, which highlights the importance of mapping potential participants’ preferences when co-designing involvement, to ensure research methods meet the needs of participants
6: Improved understanding of how to get involved in research	Participants reported improved understanding of how to get involved in research; this helped inform decision making for individuals when invitations were sent to members of the group to participate in genomics research after this study and unconnected to this study [22]. **Learning point**: Pre and post discussion surveys and standardised reporting (STARDIT-PM) are useful tools for mapping changes in understanding and views [20, 71]
7: Co-design changed study design	Feedback from participants resulted in changes to the study design including improving language used in recruitment and learning resources. **Learning point:** Involving participants in helping co-create learning resources can improve them
8: Method for future research co-design established	Participants stated that the methods used in this process could be helpful when co-designing future stages of proposed genomic research with the sibling group. **Learning point:** STARDIT can be used to map preferences and impacts from future co-design processes [20]. Learning from this process is relevant to sub-populations where people share recent ancestry such as some Indigenous populations [9, 10, 66], and sub-populations of people affected by rare diseases^67^

### Participant views about genomic research and involvement

Participants demonstrated improved understanding of the difference between participation in research and involvement in research by the end of the discussion, although initially some were confused by the distinction. One stated 'I am a strong supporter of patient involvement in medical care' and that 'involving members of the public' in genomic research was important in order to 'have their views, reactions, interpretations, questions, concerns sought, interacted with, and considered' [P11].

All six participants of the online discussion thought that anyone should be involved in research, with one saying 'everyone should have a voice not just scientists and researchers' [P5]. Another added that it is a 'good idea to involve research subjects in formulating the research questions' [P10].

Methods of involving people were discussed in detail with a number of options explored. Participants explored ideas around the purpose of research and one stated that research participants should be involved in 'agreeing purpose, parameters and methods' [P7]. All expressed concern about research for profit and those with financial interests influencing research without the public being involved.

One participant noted that being 'highly educated' was an enabler for involvement as was having a 'bit of time on their hands' [P4]. Another noted they didn't feel 'qualified' to 'comment on aspects of science itself' but felt 'strongly' that they should be involved in ethical decisions and sharing personal experiences to help inform research [P5].

One participant asked 'there will be many interested groups so which ones will be listened to?' [P4]. Another noted they felt that certain pharmaceutical companies were responsible for 'bullying', contributing to 'disinformation; ignorance and inflexibility of medical and scientific professions' [P12].

### Participant views about proposed research with sibling group

Participants were supportive of future genomics research with the sibling group. One participant stated it would be ‘worth the effort’, and another stated they ‘wholeheartedly support the involvement of the next generation’ in any future research with the sibling group and noted any study design should ensure new siblings and their offspring should be involved to ensure they can ‘become part of the research’ [P9] [P5].

In reference to future research with the sibling group one participant stated that ideally 'we would be able to exert control over the use' of data [P7]. Conversely, one participant stated in the follow-up survey that they ‘could not at all care whether my genomics are public or not. I do not see that my genome is a matter for privacy concerns’ and recognised that others may feel differently [P9].

Participants spontaneously shared their views about what possible areas of research topics could be explored in studies they could participate in and how these could be conducted. These included 'mental health' [P6] and pharmacogenomics [P4], with non-health related topics including 'career choices' and hobbies [P4]. One participant felt they should be involved in 'seeking answers to old, or not yet thought of questions' and 'looking beyond the known into a murky unknown' [P6].

### Stakeholder views about the process

Participants reported their motivation for participating in this research process was altruistic, to help researchers and the wider public. Some also recognised that their participation might directly benefit members of the sibling group itself. One participant also stated ‘it is commendable’ that ‘participatory research’ was being used, in reference to the research methods used by the study team [P9]. While online discussions were perceived as having advantages and ‘worked well’ [P7], two participants reported usability issues with the online platform.

Four participants identified specific things about the way this study was conducted that enabled their involvement. One said the entire process was ‘assiduous’ and that the ‘intent of this project’ was ‘obviously thoughtful and interesting’ [P9]. One participant said the ‘system seemed to work well’ [P7]. Another added that being used to online platforms like Loomio, or having previous experience of similar platforms and ‘used to’ that way of communicating might facilitate involvement using such a communication mode [P5].

Four participants reported specific things about the way this study was conducted that were barriers to their involvement. The pace of the discussions was mentioned as moving ‘too quickly’ with another adding ‘more time’ was needed and that the study team should ‘reconsider the pace of the research’ [P7] [P4] [P5]. A discussion about creating boundaries in the discussion revealed that some participants felt that they should avoid ‘topics which might trigger emotions which are stressful’ whereas others thought this could be viewed as ‘restrictive, even censorious’ [P7].

The study team noted a ‘critical mass’ effect in online discussions, with the pace of comments seemingly affected by the number of posts. One study team member noted the difficulty in achieving ‘the balance of being prescriptive’ (for consistency) and giving freedom to facilitators to initiate discussions and follow emergent themes. Support for the facilitator by the study team was identified as an important enabler of the research process by the facilitator. Further barriers and enablers are summarised in the accompanying STARDIT report (Additional file 2).

## Discussion

In this study we used a participatory action research process to explore the views of a group of 18 known donor-conceived siblings, with participants reporting enjoying the overall experience of the surveys and discussion forum. Online text-based discussion forums were reported as an inclusive way of involving people which was more flexible for communities which exist across time zones. Participants reported it also gave them more time to reflect on answers, learn collectively as a group and provided the freedom to ask questions and share ideas throughout the process. Participants reported that the participatory process described here appears to have resulted in members of the sibling group feeling they will have more influence over research done with them [22].

Co-designing the study ensured it was more likely to meet the needs of potential participants. Involving participants in co-designing the research process resulted in a number of improvements to the study design, including improving language used in recruitment and learning resources and co-creating acceptable online discussion boundaries. The process improved participants’ understanding about genomics and research. However, data from participants indicates that increasing the time allowed for any future discussions would help ensure the process does not move ‘too quickly’ [P7].

Participation in the process led to eight outcomes, including participants ‘widening’ their views about who should be involved in research to include more people. Participants reported changed views about the value of involvement and an improved understanding about how to be involved in genomics research. Some participants reported via the co-authored STARDIT report that learning from this process informed subsequent discussions in the sibling group about participation in research, including a proposed self-managed biobank and helping them make informed decisions about participating in genomics research.

During the online discussion, the facilitator made significantly more comments than any one participant, reflecting the level of work and engagement required to facilitate a discussion. The ‘critical mass’ effect of a certain number of posts in a discussion encouraging others to post aligns with findings from other studies which have explored participants’ hesitancy in posting in online discussion forums [65]. Only one participant used a pseudonym during online discussions, and this may reflect that the other group members felt comfortable sharing views with others in the group and trusted the security and privacy of the platform, however it is unclear why some participants logged in but did not comment.

Throughout the process, the study team was faced with ambiguous policies for the ethical involvement of people in co-designing participatory research, which hamper the degree of control that potential participants had in the research process. Members of the study team reported they felt that limitations in the ethics process affected the extent to which the sibling group could be involved in the participatory action research process. This is reported in more detail in the accompanying STARDIT report (Additional file 2). Additionally, an unplanned delay of 9 months in collecting follow-up data (related to ethics processes) may have affected the quality of data collected post-discussion, with one participant adding ‘given the lapse in time, I cannot answer’ in response to a question about their experience of participating in the online discussion [P6].

Further clarity from ethics committees and relevant organisations about the ethics of participatory action research will enhance power sharing at this crucial stage of research, with standardised reporting of data allowing direct comparison of ethical methods of involvement. One participant’s suggestion that the sibling group should form their own ethics committee to guide future research reinforces the importance of transparent ways of sharing power and control in genomics research [P6], in particular for sub-populations more likely to be exploited. This includes other sub-populations where people share recent ancestry such as some Indigenous populations [9–11, 66]; populations of people affected by rare diseases caused by similar genomic variations [67]; and other sub-populations where there is a perceived power-imbalance between researchers and potential participants.

While the focus of the online discussion was involvement in genomics research, the discussion entered additional areas. This reflected the interconnected nature of the subject, including the ethical, legal and social implications of all genomics research. The discussions thus served as an exploratory focus group, mapping participants’ views in these diverse areas. For example, four participants spontaneously raised the ‘ugly’ issue of eugenics and eugenic attitudes to genomic variations, including the distinction between perceived disability and lived-experience of having certain genomics variations [P6]. Noting the history of eugenics, participants expressed fears that genomics research would continue to be used to reinforce racism and assist with genocide. One participant cited the well-documented historical precedent of a large technology company being complicit in enabling regimes to carry out negative eugenics policies [68], and also raised concerns about contemporary and future ‘misuse’ of genomic data ‘for immigration’ [P6].

The complex ethical, legal and social implications of genomics research reinforce the importance of further research to evaluate data from discussion methods such as the ones described in this article. Evidence-informed methods are urgently required in order to inform education, debate and develop international consensus on the ethical use of data from genomics research.

### Strengths

Measuring outcomes such as ‘transformative learning’ can be challenging, but is an essential requirement for understanding processes such as involving stakeholders in research and knowledge translation [39, 69, 70]. The process followed in this study approached such challenges by collecting standardised baseline and follow-up data about views. The findings from these measures suggested that involving people in online discussions about involvement in research can change their views about who should be involved in research, including ‘widening’ such views towards support for the inclusion of a wider category of stakeholders. Similarly, using STARDIT for standardised preference mapping and reporting of involvement meant outcomes from the process could be mapped more precisely [20, 71], including outcomes beyond the date of the initial data collection. Participants reported their views and perspectives changed as a result of their participation, suggesting that online discussion facilitated collaborative learning; changes in views; and ‘transformative learning’ [39, 69, 70].

Participants reported their understanding about genomics research increased as a result of participating in the study. Data from the STARDIT report completed at the end of evaluation stage of the research process indicated that learning from being involved in online discussions informed subsequent discussions in the sibling group about participation in research, including a proposed co-managed biobank [22]. Since participating in this study, some known siblings have received invitations to participate in genomics research and share their genomes with researchers unconnected to this study. The research project described in this article helped inform their decisions to participate [22], and how they would like to be involved in shaping the future of other research with the group. In addition, the sibling group has also begun discussions within the group about future research with the group, including a self-managed biobank, with discussion informed by learning from this process [22].

Involving potential participants in the co-design of any future research is essential to ensure it is appropriate and acceptable. The importance of using STARDIT-PM for standardised preference mapping when co-designing involvement was also demonstrated by participants stating their preference for online discussion methods over face-to-face discussion or interviews. Such preference mapping helped ensure the research design met the needs of potential participants.

Providing an ‘updateable’ way to report ongoing impacts and outcomes is impossible or, at best, impractical with traditional academic publishing. Since starting the study, the number of known living siblings has now grown to 46 owing to previously unknown siblings taking direct-to-consumer DNA tests. The updatable ‘living’ STARDIT report provides a way for these additional siblings to integrate the views and preferences into future reports, and report any ongoing impacts or outcomes.

While this study was co-designed and conducted before the COVID-19 pandemic, the learning outcomes from the process (summarised in Table 6) have relevance to research disciplines beyond genomics. The methods of involving people aged 50–74 in an online discussion described here now have an unexpected relevance to many disciplines. As research projects around the world seek to involve people online in novel ways, using a tool such as STARDIT to report and evaluate such methods (including co-design) in a standardised and updateable way that works across languages is more important than ever [21]. In particular, as more people become aware of the importance of storing data according to the preferences of research participants, hosting online discussions on platforms where data is stored according to these preferences is vital to ethical research conduct [36, 37]. The detail and transparency of the methods described by this article and the accompanying STARDIT report provide a repeatable method for co-designing such discussions, using free and open-source software.

### Limitations

While 14 of the 18 known siblings were involved in either co-design or as participants, only 12 siblings gave consent to join the study, and only 6 of these participants made comments in the online discussion. Some of those who did not make comments logged-in multiple times and read comments. While the follow-up survey attempted to capture the views of all 12, only the 6 active members responded, so it is hard to understand the behaviour patterns of the 6 members who did not comment. The relatively small starting cohort (18) for this study and a smaller number of active participants (6) means that although there is useful learning from our findings, their statistical significance can only be enhanced by standardised data sharing that can then combine with results with other studies [20].

As the study was designed in parallel with other studies, some aspects of the study were inflexible during the co-design process (such as the specific open-source discussion platform that was used). Some aspects could more accurately be described as ‘co-refined’ rather than ‘co-designed’, thus limiting some areas which could be influenced by the co-design process. Using STARDIT to report which stakeholders did which tasks has attempted to overcome subjective distinctions between such terms.

In the time between the start of this research process and the publication date of this paper, there are now 46 known siblings*.* Ethical constraints limited recruitment of newly discovered siblings. Further involvement to understand views and preferences about research with the now much larger sibling group would help ensure any future participatory research meets the needs and expectations of the group.

## Conclusions

The process described here provides a replicable and practical method of involving potential participants in co-designing genomics research using online discussions, with reported positive outcomes. Co-designing the study ensured it was more likely to meet the needs of potential participants and resulted in improvements to the study design. Reporting this study using ‘Standardised Data on Initiatives (STARDIT)’ to report preferences, plan and report involvement, evaluate participatory research methods and report ‘updateable’ outcomes allows ongoing comparison with other studies. Such reporting facilitates learning from this case study and contributes to data to inform evidence-based decision making when planning future research. Learning from this study contributes to the current evidence-base used to inform future ways of involving people in genomics research. Such evidence can be applied in the context of research such as self-managed biobanks for sub-populations more likely to be exploited and other sub-populations where there is a perceived power-imbalance between researchers and potential participants.

## Supplementary Information

**Additional file 1.** Data and analysis.

**Additional file 2**. STARDIT report.

**Additional file 3**. GRIPP2 report.

## Data Availability

All relevant data has been anonymised and shared in the additional files. La Trobe University is storing all raw data according to the relevant ethics policies, and invites requests for more detailed data. The stable link version for the STARDIT reports for this article can be found here: https://www.wikidata.org/wiki/Q108618394. The ‘living’ version of the report can be found here: https://wikispore.wmflabs.org/wiki/Co-designing_genomics_research_with_donor-conceived_siblings. An archived version of the STARDIT report at the time of publication can be found here: https://web.archive.org/web/20210921055514/https://wikispore.wmflabs.org/wiki/Co-designing_genomics_research_with_donor-conceived_siblings.

## Abbreviations

STARDIT: Standardised Data on Initiatives
PiiAF: Public Involvement Impact Assessment Framework Guidance
GRIPP2: Guidance for Reporting Involvement of Patients and the Public 2
