# Supplementary material for: Co-designing genomics research with a large group of donor-conceived siblings
Source: Res Involv Engagem. 2021 Dec 16;7:89. doi: 10.1186/s40900-021-00325-7 (PMC8674833; doi:10.1186/s40900-021-00325-7)
Supplement: Supplementary file 1 — Additional file 1. Data and analysis. [file 40900_2021_325_MOESM1_ESM.pdf]

Data and Analysis: Co-designing genomics research with a large group of donor-conceived siblings

About this document

This document contains additional data relevant to the case study 'Co-designing genomics research with donor-conceived siblings'[1]. In addition it contains a more detailed description of the data sources in this case study. This document includes the preferences mapping data (STARDIT-PM), and other data about this initiative[2]. The corresponding Standardised Data on Initiatives Alpha Version (STARDIT) of the report can be found in 'Additional File 2 - STARDIT report'. The 'living' STARDIT Beta version[3] report which relates to this project can be found in the references[4].

Contents

About this document 1
Contents 1
Definitions of terms 2
Case study background and context 3
Methods 4
 Case study data collection and analysis 4
Survey questions 6
 Pre-discussion survey questions 6
 Post-discussion survey questions 7
 Facilitator survey questions 9
Learning resources 10
 Summary of Learning Resources 11
 Learning resource example: Definitions and explanations 12
Data 12
 Data sources 12
 STARDIT Preference Mapping (STARDIT- PM) 14
 Demographic information 19
 Views about who should be involved in research 20
 Participant experience 21
 Investigator shared learning group 21
Data analysis 22
 Quantitative data analysis .... **Error! Bookmark not defined.**
 Qualitative data analysis 23
References 38

Definitions of terms

We have used consistent language to describe concepts throughout this case study. The list below defines important terms used throughout.

**Involvement** – The words **'involvement'** or 'being **involved'** describe the concept of people being 'involved' in research. This is when research is carried out 'with' people rather than 'on' them.[5] 'Involvement' can also be defined as when other people aside from the research team, such as the public, patients, research participants and other stakeholders, actively contribute to the research process.[6] It is the 'active involvement' in shaping and guiding research, rather than only providing data.[7–9]
**Engaged –** participants in the online discussions are described as 'engaged' if they are reading and commenting in discussions, and 'disengaged' if they are not.
**Enablers –** enablers are things which 'facilitate' certain things happening. For example, something which enables someone to participate in research.
**Facilitator –**in this article it refers to a person facilitating online discussions. For example, "**Facilitators** shared views about **enablers** of involvement"
**Online community –** as the half-siblings discovered one another through various direct-to-consumer ancestry services, they self-created an online community which used a mailing list to an email group as the mode of communication. Participants were recruited from this online community.
**Online discussion –** the study team recruited participants to an online discussion, specifically created for this study and hosted on the secure platform Loomio.
**Participant** – a person who participated in the process of sharing views and perspectives about the genomics research, including sharing views about preferences for any future involvement.
**Participatory action research (PAR)** - is an umbrella term which describes a number of related approaches, including forms of action research which embrace a participatory philosophy. Concepts such as 'co-design', 'co-creation' and 'co-production' describe involving people in the respective tasks of designing a project or creating a learning resource, and can be considered as part of participatory action research[10]. It is a process where researchers, relevant stakeholders and sometimes the public "work together, sharing power and responsibility from the start to the end of the project"[11], including knowledge generation and translation[11].
**Potential participant** – before inviting people to become participants, it was necessary to involve a number of potential participants to help advise and plan the process.
**Shared Ancestry groups –**refers to people who have shared ancestors and have been grouped into these sub-populations by genomic researchers.
**Sibling group -** refers specifically to the community of shared interest defined by people who shared the same sperm-donor father, Bertold Wiesner.
**Stakeholder** – this term includes anyone who has a 'stake' in the research, in particular those who have important knowledge, views or perspectives that should be taken into account.[12,13] In this

paper it refers to participants, representatives, patients, parents and carers of patients, potential patients and the study team (including researchers and representatives) and the wider public.
**The study –** the study refers to the formal research described in this case study, which was overseen by the 'Ethics, Integrity and Biosafety team' team at La Trobe University and the La Trobe University Human Research Ethics Committee.
**Study team** – this process was guided by the study team, who consisted of academic researchers and members of the sibling group. The work of the study team was also advised and overseen by the 'Ethics, Integrity and Biosafety team' team at La Trobe University and the La Trobe University Human Research Ethics Committee.
**Sub-populations -** This term refers to any grouping of people below the population level. Groupings can include communities of shared interest defined by shared genetic variation. These can include groupings of people who are half-siblings.
**The process** – this term will be used to describe both the study and the co-design process which involved members of the sibling group by inviting them to share views and perspectives about genomics research, including sharing views about preferences for any future involvement.

Case study background and context

The practice of artificial insemination existed in a legal and ethical grey area for many years[14]. For example, in the UK in the 1950s, the legitimacy of children conceived from a donor father was unclear as husbands were registered as the fathers, which was legally an offence[15]. Despite the recognised 'immense social, moral and medical questions' raised by this process[14], there was no legislative oversight and the practice was self-regulated by the individuals managing clinics carrying out the procedure. Members of the UK's House of Lords suggested that artificial insemination of married women with the husband's consent be classified as adultery as late as 1954[14]. Such contemporary attitudes created potential ethical, legal and social issues for families conceiving in this way[14]. Subsequently, there was a requirement for discretion for all involved, including the identities of donors which were surrounded by 'complete secrecy'[14]. Additionally, it was the view of some doctors that parents should not know the identity of the donors as it was 'incompatible with secrecy'[15]. Accordingly, many parents were encouraged to never disclose the paternity to the offspring.

The total number of donor conceived people in the UK by 1958 was estimated to be 7500, and 100,000 in the United States[14]. One pioneering clinic mentioned in debates of the UK Parliament in the 1950s was the Dr Mary Barton's medical practice, which operated in London from the 1940s to the 1960s and was responsible for at least 433 children with Dr Mary Barton stating that she had seen 600 prospective parents between 1944 and 1954[16–18]. The Barton practice used donors from 'intelligent stock' and ruled out donors where there was 'inheritable disease on that side' or 'criminality', introducing concepts of 'the eugenic quality of the donor's stock' into the very earliest years of the practice[15]. While attempts were made to find suitable donors that were a 'match', (including parents choosing whether or not they wanted a 'Jewish' donor[15])[18], some early practitioners of artificial insemination used donors from 'a very small panel of donors'[15], often from their own immediate social circles[18,19].

One prolific donor was Dr Barton's husband, the scientist Bertold Wiesner, a consulting biologist at the Royal Northern Hospital in the 1940s[20]. According to some estimates Wiesner may have fathered up to 1000 offspring during the time the clinic was operational[21], despite a 1945 British Medical Journal paper where Barton and Wiesner stated they set an 'arbitrary limit of 100 children for each donor'[15]. In the UK, a government register of donors was proposed as early as 1949[14], but

such a register was not established until 1991[22]. Subsequently, a number of people who have discovered they are the offspring of Wiesner have discovered each other by various means in subsequent years (including using direct-to-consumer genetic testing services) and formed an online community. Some members of the group have disclosed their biological relation to Wiesner (and thus other siblings) in the public domain through media, including documentaries[19,23–25], and advocated for the rights of people who are donor conceived[26].

Methods

Case study data collection and analysis

We used case study methodology to describe our experience of involving participants in an online discussion about genomics research together with pre and post discussion surveys.

Case study selection

The selection of this case study was informed by a number of factors which were appraised by the study team using the following questions[27]:

- Was it a population of people affected by genomics research, distinct from the general public?
- Was it pragmatic – was it possible to establish a mutually trusting and effective relationship within the time and resources of the research project?
- Was the power dynamic equal and not exploitative (would the research offer participants something rather than just being passive subjects?)
- Were there conflicting or competing interests which could negatively affect the research?
- Was the proposed case study ethical (including a consideration of creating a capacity burden on populations or partner organisations)

As part of a doctorate in public health genomics exploring public involvement in genomics research, one member study team (JN) began simultaneously planning a number of groups to work with to explore this area using participatory action research methodology. Unrelated to his PhD, he bought a direct-to-consumer DNA genetic test and subsequently discovered his grandfather was Bertold Wiesner and his mother was a half-sibling over up to 1000 other people[23]. After seeking advice from relevant ethics advisors, a proto-study team was assembled and began planning how to include the sibling group in co-designing a study to explore their views about involvement in genomics research. The study team contacted a researcher who had previously worked with members of this group (MC) and invited her to join the team in order to inform study design. The study team worked closely with both potential participants and experts from the La Trobe University human ethics department to ensure the method was acceptable and no one (including the study team) would be exposed to avoidable risk.

Case study method

The case study is presented as an instrumental case study, where the purpose is to understand the particular case and can attempt to provide data that could produce useful generalisations by using inferences from the data[28]. The codesign of the case study was informed best practices for enhancing validity and rigour in the case study methodolgy[29–39]. The data collection and analysis was also informed by a number of frameworks for reporting involvement in research[13,40–44]. In addition to quantitative analysis, each source was analysed using the method of thematic analysis, which involved stages including data mapping and familiarisation, transcription, coding, searching for themes, reviewing themes with study team members, labelling and summarising themes and reporting the findings[30].

We collected and analysed both qualitative and quantitative data during the involvement activities. We also attempted to measure impacts, which can be outcomes from the participatory research process which have had an impact on individuals, the research process itself or wider society and other areas[45–47]. For example, participation in research might be shown to have a transformative impact on participants knowledge or views at an individual level, involving participants might have an impact on the research design or learning from the research may change policy or practice[2,41,48,49].

Two members of the study team were involved in analysing data from multiple sources including participant survey responses and online discussions (JN,MC). In addition, meeting notes, emails, surveys of the study team and reflexive diary entries of one member of study team (JN) were also analysed (JN). Coding and thematic analysis of qualitative data was carried out by two authors independently (JN, MC) and checked by another author (PL). Two authors of this paper also shared comments in the online study team discussion (JN, MC). Once a draft version of this article was created, it was shared with all participants who were invited to give feedback on the case study and contribute to STARDIT reports.

An alpha version of the STARDIT framework was also used in parallel with the thematic analysis to organise data into pre-defined 'super-categories' which allow consistent comparison with other data using this reporting framework[2], including other case studies.

Study team survey

The Facilitator (MC) was surveyed 6 months after the online discussion in order to integrate the valuable views and perspectives of those involved in planning and delivering the process. The survey questions can be found in the section 'Facilitator survey questions'. Design of surveys was informed by best practice frameworks for public involvement[41,44]. This method was informed by the Public Involvement Impact Assessment Framework Guidance (PiiAF)[41] and the questions were informed by sections 7 and 8 of the GRIPP2 reporting checklist[44]. The Facilitator was asked 11 questions and the data was coded and categorised, including using the STARDIT framework [2]. The data was then compared and integrated with the other data from the interviews and checked by other study team members (JN and PL).

Investigator shared learning group

During the online facilitation of the two online discussions, a shared learning group was established for Facilitators and the study teams of two similar projects being run in parallel. The study teams shared reflections and learning about the process of facilitation online, as well as offering and receiving support regarding technical and practical issues. The data was coded and categorised, including using the STARDIT framework[2].

Survey questions

Pre-discussion survey questions

After participants had read the Participant Information and given consent to participate, they were asked to complete the following information. The questions below are worded exactly as participants read them.

1. Full Name (if you would prefer to not use your real name you may use a pseudonym)
2. Email address (Please note this needs to be a working email address. If you do not have one leave this blank and we will contact you by your preferred method of communication.)
3. Phone number (optional)
4. Any other preferred method of communication? (Please share any other preferred method of communication if email or phone are not preferred)
5. Age
6. Gender (Choose from 'Male', 'Female', 'Transgender', 'Intersex', 'Other', 'Prefer not to say')
7. Educational background (tick all that apply)
    a. Middle school qualifications (up to age 16) ('lower')
    b. High school qualifications (ages 16-19) ('middle')
    c. Degree (bachelors), diploma or post-graduate ('higher')
    d. I have qualifications or professional experience in genomics (professional)
    e. Prefer not to say
8. Please tick which of the following statements that you agree with:
    a. I feel comfortable describing other descendants of my biological father as 'half-siblings'
    b. I would describe our email group as an 'online community'
    c. Members of this email group potentially have a shared interest in discussing future research which might affect them, including genomic research
    d. If you do not feel comfortable describing other descendants of your biological father as 'half-siblings' please share any term (or terms) you prefer.
9. In which country do you live (or spend most time)?
10. What made you decide to respond to our invitation to participate in this project?
11. What do you hope to get out of participating in this discussion? Do you have any specific expectations?
12. There are many benefits of involving people other than researchers in the co-design of research studies at every stage of the research cycle. Research suggests that involving people improves the quality and the relevance of the research. Involving people can also improve participant experience and increase participation. **Who do you think should influence what kind of genomic research should be done in the future?**
13. What makes you say that? (why did you give that answer?)
14. Do you have any ideas about how the people from your previous answer could influence future research? (For example, what tasks could people affected by EGID be involved in?)
15. **Which aspects of any future research genomic research should be influenced by the following (**participants were presented with a grid of tick boxes, the horizontal axis being who should be involved, the vertical a list of tasks. The horizontal was as follows)

Everyone (any member of the public who is interested)	Anyone who might be indirectly affected by the research	Only people who are directly affected by the research	Only people who are participating in the research	Only people with a professional role in research

a. All aspects mentioned below (leave others blank if ticking this)
b. Finding questions to ask (identifying research topics)
c. Deciding which questions to prioritize and fund
d. Deciding how to try and answer the question (the research method)
e. Attempting to answer the question (carrying out the research, including collecting information)
f. Trying to understand if it is possible to answer the question (analyzing the information)
g. Sharing the information that has been found, and any answers that may have emerged (dissemination and publication)
h. Ensuring that any information or answers are able to be used to help people in practice, policy or future research (sometimes called research translation)
i. Deciding if the way of asking the question and all the other stages of the research were appropriate (evaluating the research method and any impacts)
j. Designing how people are involved in the research

16. Have you ever participated in research in the past? (by participation, we mean as a research subject – for example part of a trial)
    a. Yes
    b. No
    c. Prefer not to say
    d. Unsure
17. Have you ever participated in research in the past? (by participation, we mean as a research subject – for example part of a trial)
    a. Yes
    b. No
    c. Prefer not to say
    d. Unsure

Post-discussion survey questions

1. How would you rate the following? (chose from 'Excellent', 'Somewhat good', 'Neither good nor bad', 'Somewhat poor', 'Extremely poor')
    a. Your overall experience of participating in the online discussion
    b. Your assessment of how we conducted the survey and discussion format
    c. The support you received to be involved (for example, practical support such as instructions for using the online tools)?
    d. Information and learning materials you were given before the event
2. Did you feel you meaningfully contributed to the discussion?
    a. Yes
    b. No
    c. Unsure
3. Is there anything in particular you liked or thought was helpful about how the discussion was conducted?
4. Is there anything you didn't like, thought was unhelpful. or could have been improved about how the discussion was conducted?
5. Do you have any other thoughts, ideas or comments?
6. Would you like to be updated about the progress of the research and offered chances to be involved where possible? (Choose 'yes' or 'no')
7. Did you have any expectations from participating in this research that were met or not met?

8. Have any of your views and perspectives about involving people in genomic research changed since participating in this research? If so, please describe.
9. There are many benefits of involving people other than researchers in the co-design of research studies at every stage of the research cycle. Research suggests that involving people improves the quality and the relevance of the research. Involving people can also improve participant experience and increase participation. **Who do you think should influence what kind of genomic research should be done in the future?**
10. What makes you say that? (why did you give that answer?)
11. Do you have any ideas about how the people from your previous answer could influence future research?
12. For example, what tasks could people be involved in?
13. **Which aspects of any future research genomic research should be influenced by the following (**participants were presented with a grid of tick boxes, the horizontal axis being who should be involved, the vertical a list of tasks. The horizontal was as follows)

Everyone (any member of the public who is interested)	Anyone who might be indirectly affected by the research	Only people who are directly affected by the research	Only people who are participating in the research	Only people with a professional role in research

    a. All aspects mentioned below (leave others blank if ticking this)
    b. Finding questions to ask (identifying research topics)
    c. Deciding which questions to prioritize and fund
    d. Deciding how to try and answer the question (the research method)
    e. Attempting to answer the question (carrying out the research, including collecting information)
    f. Trying to understand if it is possible to answer the question (analyzing the information)
    g. Sharing the information that has been found, and any answers that may have emerged (dissemination and publication)
    h. Ensuring that any information or answers are able to be used to help people in practice, policy or future research (sometimes called research translation)
    i. Deciding if the way of asking the question and all the other stages of the research were appropriate (evaluating the research method and any impacts)
    j. Designing how people are involved in the research
14. Full Name (Optional- if you would prefer to not use your real name you may use a pseudonym)
15. Email address (optional)
16. Phone number (optional)
17. Age
18. Gender (Choose from ‘Male’, ‘Female’, ‘Transgender’, ‘Intersex’, ‘Other’, ‘Prefer not to say’)
19. Educational background (tick all that apply)
    a. Middle school qualifications (up to age 16) ('lower')
    b. High school qualifications (ages 16-19) (‘middle’)
    c. Degree (bachelors), diploma or post-graduate ('higher')
    d. I have qualifications or professional experience in genomics (professional)
    e. Prefer not to say
20. Please tick which of the following statements that you agree with:
    a. I feel comfortable describing other descendants of my biological father as ‘half-siblings’

b. I would describe our email group as an 'online community'
c. Members of this email group potentially have a shared interest in discussing future research which might affect them, including genomic research
d. If you do not feel comfortable describing other descendants of your biological father as 'half-siblings' please share any term (or terms) you prefer.

21. In which country do you live (or spend most time)?

Facilitator survey questions

The Facilitator (MC) was surveyed 6 months after the online discussion in order to integrate the valuable views and perspectives of those involved in planning and delivering the process. Design of surveys was informed by best practice frameworks for public involvement[41,44]. This method was informed by the Public Involvement Impact Assessment Framework Guidance (PiiAF)[41] and the questions were informed by sections 7 and 8 of the GRIPP2 reporting checklist[44]. The Facilitator was asked 11 questions and the data was coded and categorised, including using the STARDIT framework [2]. The data was then compared and integrated with the other data from the interviews and checked by other study team members (JN and PL).

1. Please describe your tasks in the process of involving people in planning of the study
2. What did you learn from the process of involving participants in the research planning phase?
3. Please describe specifically what worked well or was useful about the way the study was conducted (including how people were involved)
4. Please describe specifically what did not work well or was not useful about the way the study was conducted (including how people were involved)
5. Were there any barriers or enablers to conducting the study or involvement activities? (institutional or otherwise)
6. Do you think the involvement activity achieved its intended aim(s)?
7. Do you think the study achieved its intended aim(s)?
8. Do you have any advice to other researchers planning involvement for their research?
9. Do you have any advice to other researchers planning to involve people using online discussions?
10. Describe the impact you think involving people had (positive/negative - on the research, staff or participants)
11. Who do you think should influence the kind of human genomic research done in the future, and why? (e.g. the public, participants of research studies, doctors, school children, politicians etc)
12. Which stages of future genomic research should be influenced by people other than researchers (if any)? (e.g. concept planning of new studies, study design, conducting the research, presenting the results etc)
13. Other comments

Learning resources

A number of different learning resources were shared with participants at different stages of the process. This included a short 60-second online video about the study, giving information about the context and purpose[50], a one page infographic summary of a scoping review about genomics research[51].

Learning resources were both co-created and selected by the study team, working in partnership with the Australian Genomics Health Alliance and co-refining the selection with potential participants. In addition, in order to support the facilitators in providing good quality information, a number of were curated into a list to be available for facilitators to share during the online discussion, if they became relevant to aspects of the discussion in order to help inform people. The next section summarises the resources used, with references using Internet Archive links to future-proof the content of the learning resources as well as the URL.

Summary of Learning Resources

Stage	Title	Media	Summary	Authorship
**Stage 2: Before consent**	What is genomic testing?	Portable Document Format (PDF)	Simple infographic explaining the basics of genomics research in plain English	Australian Genomics Health Alliance[52]
	What is genomics?	Online video animation with audio narration and subtitles	A 6-minute video outlining the principles of genomics research	Genome BC[53]
**Stage 3: Before online discussion**	Definitions and explanations	PDF	These definitions and explanations were used as a glossary to explain the main concepts of this research project.	Study team (see 'Learning resource example 1' below)
	Genomics and involvement	Online video with hard-coded text	A 2-minute video exploring why people should be involved in genomics	Jack Nunn[50]
	Infographic summary of scoping review	PDF (infographic – images and text)	A one page summary of the main findings from a recent scoping review about involving people in genomics[54]	Jack Nunn et al[51]
	Guide to using Loomio	PDF (text with hyperlinks)	A co-created learning resource giving practical advice for using the online discussion platform Loomio	Study team
**Additional resources available to facilitator**	Inheriting genetic conditions (chapter)	Webpage	An additional resource if participants wanted more information about inherited conditions	U.S National Library of Medicine[55]
	Data in the 100,000 Genomes Project	Online video animation with voice over and subtitles	An example of the bioinformatic pathway (specific to Genomics England) but generalisable (talks about access review committee)	Genomics England[56]
	Ethical issues in human genetics and genomics	PDF (text with hyperlinks)	Additional resource for a relevant ethics discussion from a medical perspective	Centre for Genetics Education[57]
	Genes, DNA and cancer	Webpage (text with hyperlinks)	Good plain English information about genes in relation to cancer.	Cancer Research UK[58]
	How to Share Genetic Test Results With Family	Webpage (text with hyperlinks)	Good information about sharing genetic test results with family.	American Society of Clinical Oncology[59]
	How do you sequence a human genome?	Image file (infographic – images and text)	Infographic about the stages of genome sequencing	Genomics England[60]

Learning resource example: Definitions and explanations

The following definitions and explanations were used to explain the following concepts throughout this research project. This document was formatted as a PDF with references.

Genomics

The study of all the DNA in the genome together with the technologies that allow it to be sequenced, analysed and interpreted is collectively called genomics, or genomic medicine if applied to patients[61]. The study of genomics can include other types of "omics"*, such as 'proteomics' and 'metabolomics' – which for simplicity will be referred to under the term 'genomics'. When the term 'genomics' is used in this project, it refers exclusively to human genomics.

**Other types of "Omics" include transcriptomics (all the RNA molecules in cell or organism), proteomics (all the proteins in a cell or organism) and metabolomics (all the metabolites in a cell or organism).*

Genomic research

Genomic research refers to any kind of activity which is intended to increase our current understanding of genomics. It is distinct from genomic medicine or other routine services which use existing knowledge, rather than add to it. However, this distinction is not always clear.

Involvement in genomic research

Research should be conducted ethically and to benefit people. Involving people as equal partners in genomic research has been identified as the most crucial aspect, as the benefits include improved public trust [13]. The concept of 'public involvement' in research is defined as research that is carried out 'with' people rather than 'on' them [5]. This allows people to have 'active' rather than 'passive' roles in research, which can lead to better research outcomes. Involving people in research in this way is now promoted by many governments as a right and is predicted to increasingly be an obligation in biomedical research, with research funding initiatives already recognising evidence of involvement as a key criterion [6,62,63]. Involving people can give greater public influence over research directions and conduct [64], ensuring research is both acceptable, accessible and meets people's needs by reflecting and balancing the diversity of priorities [63,65,66]. Tasks that people can be involved in include identifying areas of benefit, helping design and plan studies, helping in raising funds and analysing results.

This project

This project is part of Jack Nunn's PhD, 'Genomics research and involving people' which is focused on exploring how we can better involve people in all stages of genomics research, including the best methods to do so.

Data

Data sources

This table summarises all the data sources used for the case study.

Data Category	Data point description
**Diary**	Research diary of lead investigator (JN) – including reflections during the process
**Emails and meeting notes**	Email, meeting notes and notes from planning and discussion. This included involvement of potential participants in co-designing and co-refining the study.
**Online pre-discussion survey**	Informed consent and pre-discussion survey data
**Learning resources**	Learning resources for participants and the Facilitator giving information about genomics and using Loomio (see section ‘Learning resources’)
**Online discussion with participants**	Text data from online Loomio discussion with participants
**Online discussion with facilitators**	Text data from online Loomio discussion between Facilitators of two parallel studies
**Online post-discussion survey**	Post-discussion survey data from participants
**Follow up survey for facilitators**	Post-discussion survey data from Facilitators and additional emails with further reflections

STARDIT Preference Mapping (STARDIT- PM)

This table uses the Alpha version of the Standardised Data on Initiatives Preference Mapping (STARDIT-PM) to categorise the data into certain areas[2]. Preferences were recorded from all data sources, including the initial survey, online discussion with participants, online facilitator discussions, follow-up surveys with participants and with facilitators. Facilitator comments in the online discussion were not included. If the same participant made the same point at different stages, this was counted as one view. The standardised categorisation is intended to facilitate comparison with other studies. Accordingly, the content may be similar to other sections of the qualitative thematic analysis.

STARDIT-PM area and quantitative data	Qualitative summary
Views on who should be involved:<br><br>**10 participants** shared views about who should be involved	Participants stated that anyone should be involved in research, with a experts, people affected by the research directly and the public all sharing perspectives in the context of research carried out with ethical oversight. Six participants stated that anyone should be involved in research, with one participant stating 'everyone should have a voice not just scientists and researchers' [P5]. Another participant stated 'it needs to be a wide-ranging discussion so that the benefits and possible problems can be fully explored' [P4]. One participant said it can depend 'what kind of research it is' and what the purpose is [P7], with another adding 'we all need to have a voice' as 'we may not be "experts" in genomics but our opinions must be respected and have validity' [P5].<br><br>Six participants stated those affected by research should be involved, however others challenged this saying this could provide 'a rather one-eyed perspective' [P4]. One participant stated 'some research will benefit certain people - those people should probably influence it if they are an identifiable group'. Another participant noted that 'we're all biased; whoever is affected by a condition is likely to want it prioritised'[P12]. Another participant concluded 'we're all biased; whoever is affected by a condition is likely to want it prioritised'[P12].<br><br>One participant stated that people (including the public and research participants) will have a 'variety of professional and technical and creative skills' which will be useful, with the most useful one being 'knowing ourselves' [P7].<br><br>One participant stated 'I am a strong supporter of patient involvement in medical care' and that 'involving members of the public' in genomic research was important in order to 'have their views, reactions, interpretations, questions, concerns sought, interacted with, and considered' [P11].<br><br>Two participants stated that experts (including 'scientists'[P9]) who 'know what they are doing' should be involved [P2], with 'research reviewed by ethics boards'[P9].<br><br>There was a recognition that different groups in society might have different interests and influence. One participant articulated groups including 'medical scientists', 'social scientists', 'psychologists' and the general public as being groups which should influence research, but noted that not all 'groups should have equal influence'[P10]. One

	participant asked 'there will be many interested groups so which ones will be listened to?' [P4] One participant stated that 'people who are not looking for personal gain, but who have a desire to improve quality of life and help us understand ourselves' should influence research [P6].
Views on specific tasks people involved could do:<br><br>**8 participants** shared views about specific tasks people involved could do	One participant said that research participants should be involved in 'agreeing purpose, parameters and methods' [P7]. Another asked 'whatever format is decided upon who would decide on the points for discussion?, implying that participants' tasks should include deciding this [P4]. One participant added that it is a 'good idea to involve research subjects in formulating the research questions' [P10]. Another participant stated they should be involved in 'having a say in what research is supported by public money' and 'making sure that the uses and purposes to which the research is put are responsible and allied with the laws and mores of our society' – which includes 'ethical oversight' [P7]. One participant also added that the public should have a voice in how 'science and research can better involve' people [P5]. In reference to future research with the sibling group one participant stated that ideally 'we would be able to exert control over the use' of data [P7]. One participant felt they should be involved in 'seeking answers to old, or not yet thought of questions' and 'looking beyond the known into a murky unknown' [P6]. The discussion also explored who should be involved and in which tasks. One participant noted they didn't feel 'qualified' to 'comment on aspects of science itself' but felt 'strongly' that they should be involved in ethical decisions and sharing personal experiences to help inform research [P5]. They also stated experts 'need to drive research' but they 'cannot do it in vacuum' as the public need them and they need the public [P5].
Views on modes of communication:<br><br>**4 participants** shared views about preferred communication modes	One participant stated 'moderated face to face discussions (through video if need be) remain the best method in my opinion for focussed outcomes and decisions with groups of people' [P7}. Another participant stated that 'most of our group would be able' to use video-conferencing platforms [P5]. Face-to-face synchronous discussion was ruled out by another participant as there are 'too many voices' which are across multiple time-zones [P4]. Communicating via a verbal interview was perceived as taking less time than anything which 'requires a lot of writing' [P4]. One participant said they were 'happy to contribute in any way in which is practical online' [P5], with another adding 'email is still the best way' [P4]. One participant suggested there would be 'differing preferences for how such a project should be organised' and suggested agreeing on some ideas (not using email) and then sending these ideas to the group once decided [P4]. One participant concluded that 'a forum for considered comments' online can be useful as long as enough time is allowed [P7].
Views on what methods should be used to involve people:<br><br>**5 participants** shared views about what methods should be used to involve people	The participatory research method was described as 'commendable' [P9]. Participants suggested the idea of using one to one interviews as way of involving people (including using telecommunications) [P5], however, one participant noted that one-to-one interviews can restrict discussion and 'be quite straight jacketed with circumscribed questions', compared to online discussions [P4]. A 'group of "special interest" people involved in a group discussion' was suggested as a 'simple but effective method of encouraging debate', if participants can 'can dip in and out' [P4]. They also stated group discussion would not work as 'there are too many voices and some would be drowned out' [P4]. This participant also stated 'we will have differing preferences for how such a project should be organised' and asked 'would it be possible

	to agree on some ideas and then post them to the group' in order to involve people in co-creating how they will be involved and participate [P4].<br><br>One participant suggested that discussion, interviews, surveys and 'documents and videos shared for feedback' would all be viable methods of involving people [P5].<br><br>Another participant suggested a professional 'market researcher' who was 'tasked with finding a cross-section of people' might be a helpful method to involve people [P4].<br><br>One participant shared highly-specific views about the method that should be used, stating 'It should not be a plebiscite' nor 'self-electing moral Praetorian guard', adding that 'the more diverse the debate, the more dilute the effect of irrational preconception and ethical incompetence should become. The model of representative democracy seems to me the best available' [P8].<br><br>Another participant stated that 'ongoing discussions using social media and specific pages' such as Facebook pages, could be a good method to involve people [P5].<br><br>Another participant concluded that 'Moderated face-to-face discussions (through video if need be) remain the best method in my opinion for focussed outcomes and decisions with groups of people', adding that 'a forum for considered comments which are neither binding nor meant to be conclusive such as this can certainly be online' as long as enough time is allowed' [P7].
Views on enablers (facilitators) of involvement:<br><br>**7 participants** and **3 Facilitators** shared views about facilitators of involvement	One participant noted that being 'highly educated' was an enabler for involvement and that having a 'bit of time on their hands' was also an enabler [P4]. Being 'respectful' when involving 'those affected by genomic research' will facilitate research as the 'more brains applied to research, the more likely answer to puzzles will be found' [P11]. Similarly, another participant stated 'the more diverse the debate, the more dilute the effect of irrational preconception and ethical incompetence should become' [P8]. One participant stated that whatever model was chosen, it should be 'as flexible as possible' [P5].<br><br>Four participants reported specific things about the way this study was conducted that facilitated their involvement. One participant said the entire process was 'assiduous' and that the 'intent of this project' was 'obviously thoughtful and interesting'[P9]. One participant said the 'system seemed to work well' [P7]. Another added that being used to online platforms like Loomio, or having previous experience of similar platforms and 'used to' that way of communicating might facilitate involvement using that communication mode. One participant suggested an alternative discussion format where the participants discussed a thread for 2 days and then had a 3 day break before coming to another thread [P7].<br><br>The Facilitator (MC) noted that regular contact with the study team and timely support was essential and they 'could not have done it without this'.

Views on barriers of involvement:<br><br>**6 participants** and **3 Facilitators** shared views about barriers of involvement	Barriers to involvement in research identified by participants included public fear and 'hysteria' caused by a lack of understanding, which may 'hamper' research, involvement and general public support for research [P5]. Synchronous discussion was highlighted as another barrier if participants 'are across time zones' [P4]. One participant mentioned that they felt that their emotional response to some issues made it difficult to get involved in some ways [P6]. Being required to watch lengthy videos was identified as a barrier by one participant. One-to-one interviews were mentioned as being 'quite straight-jacketed with circumscribed questions' compared to more open online discussions [P4]. They also stated 'I don't think that a group discussion would work as there are too many voices and some would be drowned out' [P4]<br><br>Four participants reported specific things about the way this study was conducted that were barriers to their involvement. A discussion about boundaries revealed that some participants felt 'avoiding topics which might trigger emotions which are stressful or unpleasant' could be viewed as 'restrictive, even censorious' [P7]. The pace of the discussions was mentioned as moving 'too quickly' with another adding 'more time' was needed and study team should 'reconsider the pace of the research' [P7] [P4] [P5]. Updates from the discussion were sent to participants according to their preferences, and one stated they 'lost track of emails' and were sometimes unsure if they were 'responding to the right part' [P6]. Two participants stated the 'platform presented technical difficulties' [P4] and that it was 'complicated' [P5]. One participant stated the 'premise and the purpose of the study could be clearer' and that the various discussion threads were 'difficult to untangle sometimes' [P7]. They also mentioned it was 'hard to be able to guarantee to do this every day for a period' and that not doing so meant they 'got lost' [P7]. Another participant added that 'it's a difficult subject to discuss in a vacuum, without real life examples' [P4]. One participant expressed 'trepidation' at sharing views about research and compared the feeling to getting an answer wrong in an 'exam' [P6].<br><br>The Facilitator (MC) stated that they felt more time was required in the co-design process. In addition, the administrative processes surrounding the unplanned change of Chief Investigator and related administrative processes in relation to the ethics process (outside of the control of the study team) meant they felt support was 'non-existent' and was 'wholly inadequate' for the participatory research process being used.
Views on what the outcome or output of the involvement could be<br><br>**2 participants** shared views on what the outcome or output of the involvement could be	One participant stated they wanted to know that their involvement had been 'useful to the researchers' [P11], with another stating an outcome of being involved would be the 'satisfaction of knowing that I may have contributed' [P10]
Views on which stage of the research	One participant noted that changing study design in the later stages of a study not always possible, posing the question 'can we be both subjects and supervisors - at the beginning yes, later, maybe not' [P7].

people should be involved:<br><br>**1 participant** shared views about which stage of the research people should be involved	The participant also stated that compared to ‘highly-qualified scientists who know what's possible’ some ‘relatively ignorant lay people’ will be limited in what they can offer at some stages [P7]. The participant suggested that getting the ‘purposes, the parameters and the methods agreed with all participants’ at the design stage might be most appropriate [P7].
Views on who should the data from this project shared with?:<br><br>**3 participants** shared views on who data from this project should be shared with	Participants shared views about who data should be shared with, with One participant stated explicit concerns about sharing data for political or financial use [P5], with other participants agreeing. One participant stated ‘Research is for humankind. Its benefits should be available to all. Information should be for the most part easily available. If it's publicly funded, it must be publicly available [P9]’. Cultural conventions around ownership of knowledge such as ‘patenting’ were challenged as forms of knowledge control which are ‘unethical’ in some contexts.
Views on how think learning from this research could be used<br>**8 participants** shared views about how learning from this research could be used	Eight participants shared multiple views on how learning from a proposed study could be used, including predicting human traits (for example, risk of diseases or mental health problems). One participant indicated the study could help individuals in the group understand ‘what unwelcome genes we might have inherited’[P4]. Participants also indicated the research could be used to improve understanding of how things like personal experience, socioeconomic circumstances and culture interact with genomics. One participant added ‘longitudinal studies might well be set up to study a cohort of babies with certain genomic sequences which predispose them to certain diseases’ [P4]. Another participant stated research with the group ‘could have implications for all’ people [P3].

Demographic information

Category	Pre-discussion survey	Post discussion Survey
**Gender**		
Female	7	3
Male	5	3
**Age**		
50-54 years	1	0
55-59 years	2	0
60-64 years	0	0
65-69 years	6	4
70-74-years	3	2
**Educational background ('highest' only counted)**		
Degree (bachelors), diploma or post-graduate ('higher')	11	5
High school qualifications (ages 16-19)	1	1
**Number who agreed with following statements**		
I feel comfortable describing other descendants of my biological father as 'half-siblings'	12	N/A
I would describe our email group as an 'online community'	9	N/A
Members of this email group potentially have a shared interest in discussing future research which might affect them, including genomic research	11	N/A
**In which country do you live (or spend most time)?**		
Canada	3	2
Greece	1	0
Spain	1	1
United Kingdom	7	3

Views about who should be involved in research

Participants were asked the same questions before and after the online discussion. When asked who should be involved in various tasks in research, participants could choose from the categories outlined in Figure 2 in the main article. A change in direction is described as 'widening', the inverse as 'narrowing'. Widening was calculated as being a move towards an attitude that more people should be involved in research, whereas narrowing was calculated as a move towards an attitude that fewer people should be involved.

A total of 54 responses were given by 6 participants where participants completed answers to questions of both the baseline and follow-up survey. 35% of responses showed a change towards 'widening' involvement (N=19/54) while 8% 'narrowed' (N=8/54). 50% of responses stayed the same (N=27/54).

Widening and narrowing for each question

**Who should influence which aspects of research?**	**Change to wider**	**No change**	**Change to narrower**
**Finding questions to ask**	2	4	0
**Deciding which questions to prioritize and fund**	2	3	1
**Deciding how to try and answer the question** (the research method)	2	2	2
**Attempting to answer the question (carrying out the research, including collecting information)**	2	3	1
**Trying to understand if it is possible to the answer the question** (analysing the information)	3	3	0
**Sharing the information that has been found, and any answers that may have emerged** (dissemination and publication)	2	3	1
**Ensuring that any information or answers are able to be used to help people in practice, policy or future research** (sometimes called research translation)	2	3	1
**Deciding if the way of asking the question and all the other stages of the research were appropriate** (evaluating the research method and any impacts)	2	4	0
**Designing how people are involved in the research**	2	2	2
**Change totals**	19	**27**	**8**

Participant experience

How would you rate your overall experience of participating in the online discussion	
Somewhat good	4
Neither good nor bad	1
**How would you rate how we conducted the survey, and discussion format**	
Excellent	1
Somewhat good	2
Neither good nor bad	1
Somewhat poor	1
**How would you rate the support you received to be involved (for example, practical support such as instructions for using the online tools)?**	
Excellent	2
Somewhat good	2
Neither good nor bad	1
**How would you rate the information and learning materials you were given before the event**	
Excellent	3
Neither good nor bad	2
**Did you feel you meaningfully contributed to the discussion?**	
Yes	3
Unsure	3

Investigator shared learning group

During the online facilitation of the two online discussions, a shared learning group was established for facilitators and the study teams of two similar projects being run in parallel. The study teams shared reflections and learning about the process of facilitation online, as well as offering and receiving support regarding technical and practical issues. The data was coded and categorised, including using the STARDIT framework[2].

Data analysis

Quantitative analysis of all themes

Theme	Number of participants
Research for profit and ‘Bullying’ by ‘big pharma’	6
Anyone should be involved in research	6
Those affected by research should be involved	6
Research with sibling group is unique and complex but important	6
Who decides who decides what is ethical	5
Concerns about genomics research being used for political purposes	5
Finding out they are part of sibling group has been a positive experience	4
View on topics for research	4
Participants reported changed views and perspectives as a result of participating	4
Desire to improve situation for people affected by assisted conception	3
Interested in learning what other siblings think and discuss issues	3
Concerns about control of knowledge and data	3
Questioning which groups should have ‘equal influence’?	3
Questioning eugenic attitudes to genomic variations	3
Views on participation in genomics research	3
Participants learned about genomics	3
Motivation for participation to help researchers and sibling group	2
Uncertainty about what they can offer but happy to help	2
What is the purpose of research?	2
Experts should be involved (over seen by ethics boards)	2
Developments in genomics have significant implications for society	2
People have responsibility to be involved in research	1
Questioning giving power to experts	1
What control do research participants have?	1
People with specific experience and skills should be involved	1
The public and research participants should be involved in research	1
How should people be involved in genomics research	1
Tasks of involvement	1
Stages of involvement	1
Questions genomic medicine as intervention before other methods	1
Questioning genomics determinism	1
Choosing what to know about your genome	1

Qualitative data analysis

Emergent themes from qualitative participant data

This section organises qualitative data from participants into themes. This analysis has been provided in order to improve transparency of the qualitative analysis process which is summarised in the accompanying article.

Detailed Summary of Thematic Area 1: Participant views about involvement in genomic research

Participants demonstrated an understanding of the difference between participation in research and involvement in research. One participant stated 'I am a strong supporter of patient involvement in medical care' and that 'involving members of the public' in genomic research was important in order to 'have their views, reactions, interpretations, questions, concerns sought, interacted with, and considered' [P11]. Participants explored ideas around the purpose of research and one stated 'if I was the researcher running the project, I would want to get the purposes, the parameters and the methods agreed with all participants' [P7]. One participant also added that the public should have a voice in how 'science and research can better involve' people, which aligns with best-practice identified in a review of public involvement in genomics research [P5][67].

Six participants of the online discussion stated that anyone should be involved in research, with one participant stating 'everyone should have a voice not just scientists and researchers' [P5]. Another participant stated 'it needs to be a wide ranging discussion so that the benefits and possible problems can be fully explored' [P4]. One participant said it can depend 'what kind of research it is' and what the purpose is [P7], with another adding 'we all need to have a voice' as 'we may not be "experts" in genomics but our opinions must be respected and have validity' [P5].

Methods of involving people were discussed in detail with a number of options explored. Participants suggested that discussion, interviews, surveys, 'representative democracy' and 'documents and videos shared for feedback' would all be viable methods of involving people [P8] [P5]. The participatory research method was described as 'commendable' and some participants suggested the idea of using one to one interviews as way of involving people (including using telecommunications) [P9] [P5], however, one participant noted that one to one interviews can restrict discussion and 'be quite straight jacketed with circumscribed questions', compared to online discussions [P4]. This participant also stated 'we will have differing preferences for how such a project should be organised' and asked 'would it be possible to agree on some ideas and then post them to the group' in order to involve people in co-creating how they will be involved and participate [P4]. Another participant concluded that 'a forum for considered comments which are neither binding nor meant to be conclusive such as this can certainly be online' as long as enough time is allowed [P7].

Participants used a number of literary references to frame ethical debates, including citing *The Tempest* by Shakespeare as offering helpful analogies in exploring the ethics of the 'brave new world' of genomic research [P9][68], with one participant stating 'I feel more like a mix of Ariel or Caliban with respect to research - willing to help (and hoping not to be enslaved) but also willing to cede leadership' [P5]. Public fear and 'hysteria' caused by 'Brave New World fantasies' and a lack of understanding may 'hamper' involvement and general public support for research. [P9] Another participant disagreed, adding 'if we say nothing, do nothing that has to be worse doesn't it?' [P5].

One participant noted that changing study design in the later stages of a study not always possible, posing the question 'can we be both subjects and supervisors - at the beginning yes, later, maybe

not?', recognising that involvement at the design stage is the most practicable, which aligns with other similar studies [P7][69].

Six participants stated those affected by research should be involved, however others challenged this saying this could provide 'a rather one eyed perspective' [P4]. One participant stated 'some research will benefit certain people - those people should probably influence it if they are an identifiable group'. Another participant noted that 'we're all biased; whoever is affected by a condition is likely to want it prioritised' [P12]. Being 'respectful' when involving 'those affected by genomic research' will facilitate research as the 'more brains applied to research, the more likely answer to puzzles will be found' [P11]. One participant said that research participants should be involved in 'agreeing purpose, parameters and methods' [P7].

Six participants expressed concern about research for profit and those with financial interests influencing research. One participant stated they would be happy to participate in research but that they 'would however be concerned if this data was ever shared for commercial purposes' [P5]. Another participant noted they felt that certain pharmaceutical companies were responsible for 'bullying', contributing to 'disinformation; ignorance and inflexibility of medical and scientific professions' [P12]. Another participant raised concerns about 'sponsored facts' and asserted that the opinions about genomics research 'must be respected' [P5]. Another participant stated that 'the people paying for the research will influence it', noting that if it is 'public money the gatekeepers will have the greatest influence' [P7]. One participant said they find it 'distasteful that a private company can benefit from public research and then withhold the data or charge for it' [P9].

While some participants felt 'trust' towards existing scrutiny for research oversight [P5], ethics committees were identified as having 'failings', and one participant asked the question 'who will decide who will be on the ethics committee?' [P4]. Another participant added 'I am not sure of the ethics process but it does seem a shame that more of us cannot participate' [P5]. Another participant stated 'I trust the scientists and the ethical committees' [P9], while another noted that 'the ethics of DNA research generally will continue to be of huge importance and will continue as a political issue, triggering new laws and regulations' and raised a concern that 'the law will not be able to keep up with the research - and we as members of the general public won't either' [P7]. One participant noted that while people might be experts about a process or data, but that does not make them 'moral guardians' [P5]. They concluded 'no single body (and that includes the church and the government) has a right to dictate moral guidelines' [P5].

Participants indicated that all health policy decisions have political power associated with them, and five participants raised a number of specific concerns about genomic research being mis-used for political purposes and 'wicked ends' [P5]. One participant stated research should not be used 'for political purposes' but indicated they did not believe this was easily prevented [P5], with another participant sharing the 'realism about the likely corrupt use of genetic information for political and financial purposes' [P2].

Multiple participants agreed that the public can get involved in publicly funded research by overseeing what research is funded (identifying and prioritising) and ethical oversight [P7] [P5]. Three participants challenged established forms of knowledge and data control, stating that if research is 'publicly funded, it must be publicly available' and challenging concepts around intellectual property and patent laws, and that 'patenting new life forms is unethical' [P9]. Another participant stated they should be involved in 'having a say in what research is supported by public money' and 'making sure that the uses and purposes to which the research is put are responsible and allied with the laws and mores of our society' – which includes 'ethical oversight' [P7].

Views about genomics research in general

Four participants spontaneously raised the 'ugly' issue of eugenics and eugenic attitudes to genomic variations [P6] [P12]. A number of participants cited historical examples of genetic discrimination by regimes such as the Nazis as providing important learning for future genomics research. One participant cited the well documented historical precedent of the large technology company IBM being complicit in enabling regimes to carry out negative eugenics policies[70], stating IBM were 'the enablers for the Nazis ability to hunt down Jews and other "undesirables"' [P6]. The same participant also raised concerns about contemporary and future 'misuse' of genomic data 'for immigration' [P6].

Another participant raised concerns about deterministic applications of genomics for the early 'detection' of mental health problems perceived as 'undesirable' to those with political power [P7]. One participant noted that political 'demagogues' can influence public opinion and as a result they 'generally trust qualified researchers and ethics committees' and 'trust "people" far less' [P9]. Similarly, another participant stated 'the more diverse the debate, the more dilute the effect of irrational preconception and ethical incompetence should become' [P8].

Another participant raised the issue of pre-birth genetic testing, asking 'isn't it less ethical not to present informed choice', while acknowledging that the perception of impairment is subjective, and gave the example of the 'Deaf and Hearing Impaired community' not viewing deafness as 'an impairment' [P6]. The participant concluded with the question 'can we speak for those who are different to us?' [P6]. Another participant responded that 'The ethics of pre birth testing will never be clear cut' and cited the example of deaf couples to selecting 'an embryo for IVF that would be deaf like them' [P4][71].

There was a recognition that different groups in society might have different interests and influence. The discussion also explored who should be involved and in which tasks. One participant articulated groups including 'medical scientists', 'social scientists', 'psychologists' and the general public as being groups which should influence research, but noted that not all 'groups should have equal influence'[P10]. One participant noted they didn't feel 'qualified' to 'comment on aspects of science itself' but felt 'strongly' that they should be involved in ethical decisions and sharing personal experiences to help inform research [P5]. One participant asked 'there will be many interested groups so which ones will be listened to?' [P4].

One participant noted that being 'highly educated' was an enabler for involvement and that having a 'bit of time on their hands' was also a enabler [P4].

Detailed Summary of Thematic Area 2: Participant views about proposed research with sibling group

Participants recognised they were part of a 'unusual cohort' and suggested they would be useful to study [P4]. One participant stated they believed the sibling group 'were part of a eugenics programme in some way' [P4]. Participants shared multiple views about proposed research with the sibling group. One participant stated that 'this field of study is so huge and our involvement would be a 'first' in many ways' [P5]. Participants recognised the complexity of research on their 'community of shared interest' owing to multiple variables and one participant stated research with the sibling group would be 'enormously complicated' owing to 'confounding variables' but would be 'worth the effort' [P9]. Another participant added that they would 'wholeheartedly support the involvement of the next generation' in any future research with the sibling group and noted any study design should ensure new siblings and their offspring should be ensure they can 'become part of the research' [P5] .

One participant noted ‘there is a world of difference between the idea of a study’ of the sibling group and other genomic research [P7]. Another participant concluded that ‘we are on the cusp of new forms of information , study and knowledge about ourselves’ and stated that in the context of genomic research ‘we are important in the grand scheme of things’ [P6]. In a follow-up survey one participant stated they ‘could not at all care whether my genomics are public or not. I do not see that my genome is a matter for privacy concerns’ but recognised that others may feel differently [P9].

One participant proposed that research with the sibling group would ‘not be trying to push a set agenda or profit financially’ [P5]. Another stated that ‘people who are not looking for personal gain’ should be involved, including those ‘who have a desire to improve quality of life and help us understand ourselves’ [P6]. One participant added that it is a ‘good idea to involve research subjects in formulating the research questions’ [P10]. Another asked ‘whatever format is decided upon who would decide on the points for discussion?, implying participants’ tasks should include deciding this [P4]. One participant suggested that members of the sibling group could ‘form and seek out participants for the Ethics Committee’ [P6].

One participant stated it was a ‘civic duty’ to participate in research and that research participants should be involved in formulating the research questions [P10]. Participants spontaneously suggested potential research topics for the group, some serious issues and some appeared to be more light-hearted comments made in jest. More serious topics included ‘mental health’ [P6] and pharmacogenomics [P4], with more light-hearted suggestions including ‘career choices’ and hobbies [P4]. One participant felt they should be involved in ‘seeking answers to old, or not yet thought of questions’ and ‘looking beyond the known into a murky unknown’ [P6].

Participants shared multiple views about what possible areas of research topics could be explored in studies they could participate in and how these could be conducted Participants spontaneously suggested potential research topics for the group, some serious and some more light-hearted comments made in jest. More serious topics included ‘mental health’ [P6] and pharmacogenomics [P4], with more light-hearted suggestions including ‘career choices’ and hobbies [P4]. One participant felt they should be involved in ‘seeking answers to old, or not yet thought of questions’ and ‘looking beyond the known into a murky unknown’ [P6].

One participant suggested genomics research ‘a subject which cries out for more public discussion by those who have been unexpectedly and deeply affected by genomics findings’ [P5]. Another participant suggested a study of ‘any intersection of phenotypes’ including ‘psychological and intellectual’ with other genomics data [P8]. The participant concluded with the question ‘I have wondered what unwelcome genes we might have inherited?’, citing their own experience of genetic testing for both Parkinson’s and Huntington’s disease [P4].

One participant reported they were ‘happy to contribute any information from my own genome that might be useful in scientific or forensic research’ [P9]. One participant suggested a study design where participants can ‘all participate or not as we wish’ [P5]. They also commented they were ‘OK to provide genetic material for further analysis’. [P5]. Another participant concluded the siblings should ‘contribute their DNA’ for research [P9] and warned people from thinking that ‘your DNA is “you”’ [P9].

A ‘group of “special interest” people involved in a group discussion’ was suggested by one participant as a ‘simple but effective method of encouraging debate’, if participants can ‘can dip in and out’ [P4]. They also stated group discussion would not work as ‘there are too many voices and some would be drowned out’ [P4]. This participant also stated ‘we will have differing preferences for how such a project should be organised’ and asked ‘would it be possible to agree on some ideas and

then post them to the group' in order to involve people in co-creating how they will be involved and participate [P4].

The participatory research method was described as 'commendable' [P9]. One participant stated 'moderated face to face discussions (through video if need be) remain the best method in my opinion for focussed outcomes and decisions with groups of people' [P7}. Another participant stated that 'most of our group would be able' to use video-conferencing platforms [P5]. Face to face synchronous discussion was ruled out by another participant as there are 'too many voices' which are across multiple time-zones [P4]. Participants suggested the idea of using one to one interviews as way of involving people (including using telecommunications) [P5], however, one participant noted that one to one interviews can restrict discussion and 'be quite straight jacketed with circumscribed questions', compared to online discussions [P4].

In reference to future research with the sibling group one participant stated that ideally 'we would be able to exert control over the use' of data [P7].

Detailed Summary of Thematic Area 3: Participant views about the online discussion

Participants reported their motivation for participating in the research was to help researchers and the sibling group. Three participants stated they were participating partly because they were interested in learning what their siblings think, and the opportunity to 'think through' and 'discuss' issues together [P4] [P6].

Participants were asked to comment on their experience of participating in this study. One participant mentioned they were 'surprised about the number of participants' in the study, stating they thought more siblings would have participated [P6]. Others stated the experience was 'interesting' and they 'enjoyed thinking about the questions posed and reading the responses of others' and the 'perceptive comments' of the Facilitator [P7] [P4]. One participant stated 'it is commendable that there is a concern about participatory research' in reference to the research methods used by the study team [P9]. However the participant also noted that in the early stages of participatory action research there was more discussion than action [P9].

Four participants reported specific things about the way this study was conducted that facilitated their involvement. One participant said the entire process was 'assiduous' and that the 'intent of this project' was 'obviously thoughtful and interesting'[P9]. One participant said the 'system seemed to work well' [P7]. Another added that being used to online platforms like Loomio, or having previous experience of similar platforms and 'used to' that way of communicating might facilitate involvement using that communication mode. One participant suggested an alternative discussion format where the participants discussed a thread for 2 days and then had a 3 day break before coming to another thread [P7].

Four participants reported specific things about the way this study was conducted that were barriers to their involvement. A discussion about boundaries revealed that some participants felt 'avoiding topics which might trigger emotions which are stressful or unpleasant' could be viewed as 'restrictive, even censorious' [P7]. The pace of the discussions was mentioned as moving 'too quickly' with another adding 'more time' was needed and the study team should 'reconsider the pace of the research' [P7] [P4] [P5]. Two participants stated the 'platform presented technical difficulties' [P4] and that it was 'complicated' [P5]. One participant stated the 'premise and the purpose of the study could be clearer' and that the various discussion threads were 'difficult to untangle sometimes' [P7]. They also mentioned it was 'hard to be able to guarantee to do this every

day for a period' and that not doing so meant they 'got lost' [P7]. Another participant added that 'it's a difficult subject to discuss in a vacuum, without real life examples' [P4].

Detailed summary of all other thematic areas

Finding out they are part of sibling group has been a positive experience

The study team also noted that before the main discussion began, participants shared many personal experiences and reflections on finding out they were part of the extended family. For example four participants spontaneously reported that finding out they had siblings and being part of the siblings' online community was a positive experience, with one considering themselves 'lucky' [P4]. Another participant added 'I am so happy to be part of this [group]' and that the experience 'has changed and enriched my life' [P6]. They added 'we are beyond fortunate, we have our group' [P6]. Participants' reactions also suggested the siblings group offered a form of support, and are in contrast to contemporary views of the 1950s where people feared that offspring finding out about their origins might cause 'psychological injury'[14].

People have responsibility to be involved in research

One participant stated it was a 'civic duty' to participate in research and that research participants should be involved in formulating the research questions [P10].

Motivation for participation to help researchers and sibling group

Participants reported their motivation for participating in the research was to help researchers and the sibling group.

Uncertainty about what they can offer but happy to help

Two participants stated that while they were happy to help with research, they were uncertain about what they could offer or did not believe they were 'knowledgeable enough' [P6].

Desire to improve situation for people affected by assisted conception

Three participants stated they hoped future research would improve the situation for people affected by donor conception, as 'the views of donor conceived people were not considered for many years' [P4]. One participant stated 'there should be better and accessible structures in place to support people through the process of self-discovery' [P5].

Interested in learning what other siblings think and discuss issues

Three participants stated they were participating partly because they were interested in learning what their siblings think, and the opportunity to 'think through' and 'discuss' issues together [P4] [P6].

Concerns about power imbalance in research

Seven participants shared multiple views about power imbalances in research, including concerns about bias, conflicting interests, data breaches and 'hidden motives' [P2], with genomic research being 'used to perverted ends' for both political and financial purposes [P2].

Control of knowledge and data

Three participants challenged established forms of knowledge and data control, stating that if research is 'publicly funded, it must be publicly available' and challenging concepts around intellectual property and patent laws, and that 'patenting new life forms is unethical' [P9]. Another

participant raised concerns about ‘sponsored facts’ and asserted that the opinions about genomics research ‘must be respected’ [P5].

Who decides who decides what is ethical?

While some participants felt ‘trust’ towards existing scrutiny for research oversight [P5], ethics committees were identified as having ‘failings’, and one participant asked the question ‘who will decide who will be on the ethics committee?’ [P4]. One participant suggested that members of the sibling group could ‘form and seek out participants for the Ethics Committee’ [P6]. Another participant added ‘I am not sure of the ethics process but it does seem a shame that more of us cannot participate’ [P5]. Another participant stated ‘I trust the scientists and the ethical committees’ [P9], while another noted that ‘the ethics of DNA research generally will continue to be of huge importance and will continue as a political issue, triggering new laws and regulations’ and raised a concern that ‘the law will not be able to keep up with the research - and we as members of the general public won't either’ [P7]. One participant noted that while people might be experts about a process or data, but that does not make them ‘moral guardians’ [P5]. They concluded ‘no single body (and that includes the church and the government) has a right to dictate moral guidelines’[P5].

Genomics research used for political purposes

While some participants indicated that all health policy decisions have political power associated with them, five participants raised a number of specific concerns about genomic research being mis-used for political purposes and ‘wicked ends’ [P5]. One participant stated research should not be used ‘for political purposes’ but indicated they did not believe this was easily prevented [P5], with another participant sharing the ‘realism about the likely corrupt use of genetic information for political and financial purposes’[P2]. A number of participants cited historical examples of genetic discrimination by regimes such as the Nazis as providing important learning for future genomics research. One participant cited the well documented historical precedent of the large technology company IBM being complicit in enabling regimes to carry out negative eugenics policies[70], stating IBM were ‘the enablers for the Nazis ability to hunt down Jews and other "undesirables"’ [P6]. The same participant also raised concerns about contemporary and future ‘misuse’ of genomic data ‘for immigration’ [P6]. Another participant raised concerns about deterministic applications of genomics for the early ‘detection’ of mental health problems perceived as ‘undesirable’ to those with political power [P7].

Questioning giving power to experts ‘reinforces dependency on experts’

One participant noted that ‘blind fear and reinforces dependency on experts, at the expense of genuinely holistic solutions’ and that the people should challenge the ‘unquestioning faith in a kind of scientific determinism’ which genomics can encourage [P12]. Another participant felt experts ‘need to drive research’ but they ‘cannot do it in vacuum’ as the public need them and they need the public [P5].

Research for profit and ‘Bullying’ by ‘big pharma’

Six participants expressed concern about research for profit and those with financial interests influencing research. One participant stated they would be happy to participate in research but that they ‘would however be concerned if this data was ever shared for commercial purposes [P5]. Another participant noted they felt that certain pharmaceutical companies were responsible for ‘bullying’, contributing to ‘disinformation; ignorance and inflexibility of medical and scientific professions’ [P12]. One participant proposed that research with the sibling group would ‘not be trying to push a set agenda or profit financially’ [P5]. Another stated that ‘people who are not looking for personal gain’ should be involved, including those ‘who have a desire to improve quality of life and help us understand ourselves’ [P6]. Another participant stated that ‘the people paying for the research will influence it’, noting that if it is 'public money the gatekeepers will have the greatest influence’ [P7]. One participant said they find it ‘distasteful that a private company can benefit from public research and then withhold the data or charge for it’ [P9].

What control do participants have?

One participant raised the question of 'what type of control the subjects of the study were allowed' [P7]. They posed the question 'Can we be both subjects and supervisors' and suggested that this would be easier at the start during the design phase but more challenging later on in the research cycle [P7].

Why do we do research?

One participant stated that research is 'for humankind' [P9], with another going further and saying 'research is for every and any aspect of life', including other forms of life [P5].

Who should be involved in research

Participants stated that anyone should be involved in research, with a experts, people affected by the research directly and the public all sharing perspectives in the context of research carried out with ethical oversight.

Anyone should be involved in research

Six participants stated that anyone should be involved in research, with one participant stating 'everyone should have a voice not just scientists and researchers' [P5]. Another participant stated 'it needs to be a wide ranging discussion so that the benefits and possible problems can be fully explored' [P4]. One participant said it can depend 'what kind of research it is' and what the purpose is [P7], with another adding 'we all need to have a voice' as 'we may not be "experts" in genomics but our opinions must be respected and have validity' [P5].

Those affected by research should be involved

Six participants stated those affected by research should be involved, however others challenged this saying this could provide 'a rather one eyed perspective' [P4]. One participant stated 'some research will benefit certain people - those people should probably influence it if they are an identifiable group'. Another participant noted that 'we're all biased; whoever is affected by a condition is likely to want it prioritised'[P12].

People with specific experience and skills should be involved

One participant stated that people (including the public and research participants) will have a 'variety of professional and technical and creative skills' which will be useful, with the most useful one being 'knowing ourselves' [P7].

The public and research participants should be involved in research

One participant stated 'I am a strong supporter of patient involvement in medical care' and that 'involving members of the public' in genomic research was important in order to 'have their views, reactions, interpretations, questions, concerns sought, interacted with, and considered' [P11].

Experts should be involved (over seen by ethics boards)

Two participants stated that experts (including 'scientists'[P9]) who 'know what they are doing' should be involved [P2], with 'research reviewed by ethics boards'[P9]. One participant stated they 'loathe this current political atmosphere that is anti-intellectual, anti-expert, anti-science' and that is why they believe that genomics research should be 'left to the qualified' [P9]. This view was challenged by another participant who stated that it was often people who think they are qualified who in fact sometimes know 'nothing at all', for example regarding personal experiences 'in practical terms' [P5].

Which groups should have 'equal influence'?

There was a recognition that different groups in society might have different interests and influence. The discussion also explored who should be involved and in which tasks. One participant articulated groups including 'medical scientists', 'social scientists', 'psychologists' and the general public as

being groups which should influence research, but noted that not all 'groups should have equal influence'[P10]. One participant noted they didn't feel 'qualified' to 'comment on aspects of science itself' but felt 'strongly' that they should be involved in ethical decisions and sharing personal experiences to help inform research [P5]. One participant asked 'there will be many interested groups so which ones will be listened to? adding that 'It is easy to say only those people who are directly affected should have an influence on research but theirs could be a rather one eyed perspective' [P4].' [P4] One participant stated that 'people who are not looking for personal gain, but who have a desire to improve quality of life and help us understand ourselves' should influence research [P6].

Views on genomics research

Participants shared views on genomics which demonstrated a high-level of medical and genomic literacy. One participant who is a qualified medical professional stated developments in genomics have 'been the most significant of my lifetime' [P8].

Another participant questioned the use of genomic medicine as an intervention before other solutions such as 'simple lifestyle changes' [P12]. They also questioned 'a kind of scientific determinism' which genomic research can encourage.

Eugenics

Four participants spontaneously raised the 'ugly' issue of eugenics and eugenic attitudes to genomic variations [P6] [P12]. One participant stated they believed the sibling group 'were part of a eugenics programme in some way' [P4]. Another participant raised the issue of pre-birth genetic testing, asking 'isn't it less ethical not to present informed choice', while acknowledging that the perception of impairment is subjective, and gave the example of the 'Deaf and Hearing Impaired community' not viewing deafness as 'an impairment' [P6]. The participant concluded with the question 'can we speak for those who are different to us?' [P6]. Another participant responded that 'The ethics of pre birth testing will never be clear cut' and cited the example of deaf couples to selecting 'an embryo for IVF that would be deaf like them' [P4][72]. The participant concluded with the question 'I have wondered what unwelcome genes we might have inherited?', citing their own experience of genetic testing for both Parkinson's and Huntington's disease [P4].

Views on participation in genomics research

One participant suggested a study design where participants can 'all participate or not as we wish' [P5]. They also commented they were 'OK to provide genetic material for further analysis'. [P5]. Another participant concluded the siblings should 'contribute their DNA' for research [P9] and warned people from thinking that 'your DNA is "you"' [P9].

Research with siblings is unique and complex but important

One participant stated research with the sibling group would be 'enormously complicated' owing to 'confounding variables' but would be 'worth the effort' [P9]. Another participant added that they would 'wholeheartedly support the involvement of the next generation' in any future research with the sibling group and noted any study design should ensure new siblings and their offspring should be ensure they can 'become part of the research' [P5] . On participant noted 'there is a world of difference between the idea of a study' of the sibling group and other genomic research [P7]. Another participant concluded that 'we are on the cusp of new forms of information , study and knowledge about ourselves' and stated that in the context of genomic research. 'we are important in the grand scheme of things' [P6]. In a follow-up survey one participant stated the 'could not at all

care whether my genomics are public or not. I do not see that my genome is a matter for privacy concerns' but recognised that others may feel differently [P9].

Research topics – 'into a murky unknown'

Participants spontaneously suggested potential research topics for the group, some serious and some more light-hearted comments made in jest. More serious topics included 'mental health' [P6] and pharmacogenomics [P4], with more light-hearted suggestions including 'career choices' and hobbies [P4]. One participant felt they should be involved in 'seeking answers to old, or not yet thought of questions' and 'looking beyond the known into a murky unknown' [P6].

One participant suggested genomics research 'a subject which cries out for more public discussion by those who have been unexpectedly and deeply affected by genomics findings' [P5]. Another participant suggested a study of 'any intersection of phenotypes' including 'psychological and intellectual' with other genomics data [P8]. One participant also indicated that involvement in research might help people make sense of their personal experience and added joining an online discussion 'will let me think beyond my emotion'[P6].

Choosing what to know about your genome

Participants shared views which recognised there might be important variations in the knowledge people might chose to have about themselves and their genomes. One participant stated knowledge about whether someone was carrying a variation which pre-disposed them to Huntington's could be a 'poisoned chalice' [P4].

Co-creating discussion boundaries

In addition to inviting potential participants to help co-design discussions, participants were themselves invited to co-create their own boundaries for the group discussion by reviewing a suggested statement and suggesting amendments. The group was invited to self-creates code of conduct based on previous negative experiences of people unintentionally causing offence. One participant stated there was difficulty in self-censoring and knowing what others might find 'emotionally stressful or unpleasant' [P7]. One participant stated 'Common courtesy should be sufficient' [P2]Another participant suggested 'Perhaps we might agree that if one of us unintentionally makes a comment perceived as offensive by another that we agree either to apologise or explain our position' [P5].

Experience of participating in research

Participants were asked to comment on their experience of participating in this study. One participant mentioned they were 'surprised about the number of participants' in the study, stating they thought more siblings would have participated [P6]. Others stated the experience was 'interesting' and they 'enjoyed thinking about the questions posed and reading the responses of others' and the 'perceptive comments' of the Facilitator [P7] [P4]. One participant stated 'it is commendable that there is a concern about participatory research' in reference to the research methods used by the study team [P9]. However the participant also noted that in the early stages of participatory action research there was more discussion than action [P9].

Enablers and barriers specific to this study

Participants reported a number of enablers and barriers for involvement which were specific to this study.

Enablers of participation and involvement

Four participants reported specific things about the way this study was conducted that facilitated their involvement. One participant said the entire process was 'assiduous' and that the 'intent of this project' was 'obviously thoughtful and interesting'[P9]. One participant said the 'system seemed to work well' [P7]. Another added that being used to online platforms like Loomio, or having previous experience of similar platforms and 'used to' that way of communicating might facilitate involvement using that communication mode. One participant suggested an alternative discussion format where the participants discussed a thread for 2 days and then had a 3 day break before coming to another thread [P7]. The Facilitator (MC) noted that regular contact with the study team and timely support was essential and they 'could not have done it without this'.

Barriers of participation and involvement

Four participants reported specific things about the way this study was conducted that were barriers to their involvement. A discussion about boundaries revealed that some participants felt 'avoiding topics which might trigger emotions which are stressful or unpleasant' could be viewed as 'restrictive, even censorious' [P7]. The pace of the discussions was mentioned as moving 'too quickly' with another adding 'more time' was needed and the study team should 'reconsider the pace of the research' [P7] [P4] [P5]. Updates from the discussion were sent to participants according to their preferences, and one stated they 'lost track of emails' and were sometimes unsure if they were 'responding to the right part' [P6]. Two participants stated the 'platform presented technical difficulties' [P4] and that it was 'complicated' [P5]. One participant stated the 'premise and the purpose of the study could be clearer' and that the various discussion threads were 'difficult to untangle sometimes' [P7]. They also mentioned it was 'hard to be able to guarantee to do this every day for a period' and that not doing so meant they 'got lost' [P7]. Another participant added that 'it's a difficult subject to discuss in a vacuum, without real life examples' [P4]. One participant expressed 'trepidation' at sharing views about research and compared the feeling to getting an answer wrong in an 'exam' [P6].

The Facilitator (MC) stated that they felt more time was required in the co-design process. In addition, the administrative processes surrounding the unplanned change of Chief Investigator and related administrative processes in relation to the ethics process (outside of the control of the study team) meant they felt support was 'non-existent' and was 'wholly inadequate' for the participatory research process being used.

Emergent themes from qualitative investigator discussion data

A number of themes were identified during a qualitative thematic analysis of the discussion. In order to maintain confidentiality, comments have not been attributed to individual Facilitators and have been shared with the permission of those who participated in discussions.

A personal or professional perspective?

Facilitators reported finding it a challenge to separate 'personal' experiences from 'professional perspectives' when facilitating. The platform was new to all Facilitators and a considerable amount of time was required in order to both train and support Facilitators using the platform. While some issues were platform specific, any such platform will require training and ongoing support for those new to using it. This includes real-time video and voice calls.

Facilitators required guidance on using personal experience, with the confidential investigator group serving as a place for advice and support. Future research of this kind should ensure that facilitators are supported appropriately, including being part of an active and confidential community of practice in order to give and receive practical, emotional and technical support.

One members of the study team noted that facilitation is ‘not a dispassionate neutral and purely intellectual’ activity, and that some kind of personal motivation for facilitation can be helpful. This aligns with findings from the online discussion with participants, where the personal experience of being part of the sibling group was hard to separate from general views about genomics research.

When analysing the data, one Facilitator noted ‘subjectivity isn't something shameful to be avoided - it's something to be acknowledged’. They later added:

> ‘I think this discussion has also explored the boundaries of 'researcher'/'neutral Facilitator'/'person with skin in the game' - stepping back, they are all social constructions - some defined by law/ethics - others by un-codified conventions - and labelling them all is quite a liberating process.’

Critical mass

Facilitators noted a ‘critical mass’ effect in online discussions, with the pace of comments seemingly affected by number of posts. The rate of posts to a discussion would increase exponentially, with more comments seeming to generate more comments in a ‘snow-ball’ effect. Conversely, discussions with fewer comments and infrequent posts attracted fewer replies, with ‘energy levels’ appearing to ‘drop’. Facilitators theorised people were more confident to post in forums if there were more people posting, so they were less ‘exposed’. This aligns with findings from other studies which have explored participants’ hesitancy in posting in online discussion forums[73].

Discussion pacing and participant engagement

When co-designing discussions, the study team attempted to balance discussion pacing between being too slow and losing engagement and being too fast and overwhelming participants. After the discussions one Facilitator reflected they feared that ‘introducing a new thread might interrupt the flow’ and that it was difficult to judge this using this with online discussions.

Facilitators also reported struggling to balance keeping people engaged and trying to get hesitant participants (people logging in, reading, but not commenting) to comment. Similarly, some active participants in discussions would not post for a number of days. Follow-up survey data from participants suggested that sometimes they could not post owing to other commitments, but the nature of online discussions meant they could catch up and join in when they had the time.

One Facilitator noted that they underestimated how long it would take for people to get ‘properly signed up’, including giving technical support to participants. This created a tension when they ‘wanted the latecomers to have time to 'catch up' without being overwhelmed’. Another Facilitator also commented that they felt it took a week for trust to be established in the group, for example, for people to see that the co-created rules of the discussion were observed by everyone.

Another Facilitator noted that:

> ‘an advantage of this kind of discussion is that you can have those simultaneous discussions - yes people have a finite read/write/processing time in a day - but it allows those with interests in specific areas to pursue that without feeling left out, as they might do with a more traditional linear time discussion (like face to face or synchronous webinars)’

They also noted it allowed people to ‘refer back’ to previous discussions, making them more rich. Participants also reported enjoying having time to reflect on comments and reconsider views in light of others’ comments.

Prescribed discussion and open-ended discussion

One investigator noted the difficulty in achieving 'the balance of being prescriptive (for consistency) and giving freedom to Facilitators' to initiate discussions and follow emergent themes. Participants reported that they enjoyed being able to raise issues and that this was preferable over the more traditional scripted interviews, as it allowed areas to be discussed that researchers might not have considered. Facilitators agreed that using the 'forking' function on Loomio to 'fork' discussions moving in different directions into threads should be avoided as might confuse both participants and Facilitators.

Ethical limitations for participatory research

The entire study team agreed that limitations in the ethics process affected the extent of how the sibling group could be involved in the study. Internationally, confusion still surrounds what ethical approval is required before involving potential participants in co-designing research as 'specialist advisors'[74], with some guidelines now emmerging[75]. On the advice of the La Trobe University Human Research Ethics Committee, we did not approach potential participants about co-designing the study until after ethics approval had been granted, with feedback from participants being incorporated by a number of subsequent modifications to the original ethics application. As a result of the complex process of modifications, the timeline for feedback was shorter than the study team had anticipated, although the process did provide useful feedback[75]. Ambiguous policies for the ethical involvement of people in co-designing research can hamper the degree of control potential participants have in research and further clarity from ethics committees will enhance power sharing at this crucial stage of research.

Impacts from the process: Detailed summary

Eight specific impacts were reported from this process. These are summarised in detail below.

Impact 1: Improved understanding of genomics

While participants showed a good-baseline level understanding of genomics, three reported their understanding about genomics and research increased as a result of participating in the study. However some participants demonstrated a lack of self-confidence in their understanding, in spite of demonstrating a good understanding of the principles of genomic research, citing relevant peer-reviewed literature in discussions and discussing the nuances of ethical oversight.

Impact 2: Learning resources useful

Participants reported finding the information resources and videos useful. One participant commented they had learned from the visual summary of the review of public involvement in genomic research stating 'I didn't realise there were so many research projects involving global genomics projects but it is so good to read that public involvement is becoming more and more important' [P5] However one participant stated 'I must say that personally I hate being required to watch videos but think I am in a minority' [P4].

Impact 3: Changed views and perspectives as a result of participating

Four out of the six participants who completed the follow up survey noted their views and perspectives changed as a result of participating. One participant stated 'I now realise how fast the field of genomics is changing and there are all kinds of implications especially in the field of precision medicine' [P5]. One participant also indicated that involvement in research might help people make sense of their personal experience and added joining an online discussion 'let me think beyond my emotion' [P6]. Another stated 'involving people in genomic research is crucial' as it has 'unknown consequences and needs as wider discussion as possible'[P4]. A number of participants had views about 'leaving research to the qualified' challenged by other participants [P9], with follow-up survey data suggesting that those challenged changed their views about who should be involved, towards widening. The changed views of the participants involved can be viewed as an impact of 'transformative learning' [76].

Impact 4:Participants asked to stay involved in the research

All participants who completed the follow-up survey requested to stay involved in the research process, including in analysing data and being co-authors on the paper.

Impact 5: Participants enjoyed the online discussions

Participants stated the experience of participating was 'interesting' and they 'enjoyed thinking about the questions posed and reading the responses of others' and the 'perceptive comments' of the Facilitator [P7] [P4]. Another participant added that it 'work well' as a way of involving people [P7].

Impact 6: Improved understanding of how to get involved in research

Participants reported improved understanding of how to get involved in research. However some participants demonstrated a lack of self-confidence in their usefulness in being involved in genomics research. Learning and development interventions to help people understand the valuable tasks they can complete in the research process might improve confidence.

Impact 7: Co-design changed study design

Feedback from participants resulted in changes to the study design including improving language used in recruitment, improving the online discussions and learning resources.

Impact 8: Method for future research co-design established

By co-creating methods of involving participants in proposed future genomics research, this process has demonstrated a practical and well-evaluated method of involving potential participants in co-designing research. Participants stated that the methods used in this process could be helpful when co-designing future stages of proposed genomic research with the sibling group.

References

1. Nunn J, Crawshaw M, Lacaze P. *Co-Designing Genomics Research With A Large Group of Donor-Conceived Siblings*.; 2020. doi:10.21203/rs.3.rs-100595/v1

2. Nunn J, Shafee T, Chang S, et al. Standardised Data on Initiatives - STARDIT: Alpha Version. 2019. doi:10.31219/osf.io/5q47h

3. Nunn JS, Shafee T. Standardised Data on Initiatives – STARDIT: Beta Version. doi:10.31219/osf.io/w5xj6

4. Nunn JS, Crawshaw M, Lacaze P, et al. *Co-Designing Genomics Research with Donor-Conceived Siblings (STARDIT Beta Version Report)*. https://www.wikidata.org/wiki/Q108618394. Accessed September 7, 2021.

5. National Institute for Health Research. Briefing note eight: Ways that people can be involved in the research cycle. National Institute for Health Research. http://web.archive.org/web/20170605035051/http://www.invo.org.uk/posttyperesource/where-and-how-to-involve-in-the-research-cycle/. Published 2017. Accessed June 5, 2017.

6. National Institute for Health Research. Patient and public involvement in health and social care research. 2014. https://www.nihr.ac.uk/about-us/CCF/funding/how-we-can-help-you/RDS-PPI-Handbook-2014-v8-FINAL.pdf. Accessed February 2, 2018.

7. Oliver S, Clarke-Jones L, Rees R, et al. Involving consumers in research and development agenda setting for the NHS: Developing an evidence-based approach. *Health Technol Assess (Rockv)*. 2004;8(15):1-148, III-IV. doi:10.3310/hta8150

8. Staniszewska S, Adebajo A, Barber R, et al. Developing the evidence base of patient and public involvement in health and social care research: the case for measuring impact. *Int J Consum Stud*. 2011;35(6):628-632. doi:10.1111/j.1470-6431.2011.01020.x

9. International Association for Public Participation. Participation Spectrum. https://www.iap2.org.au/Tenant/C0000004/00000001/files/IAP2_Public_Participation_Spectrum.pdf. Published 2014. Accessed July 12, 2018.

10. Macaulay AC. Participatory research: What is the history? Has the purpose changed? *Fam Pract*. 2016;351(3):cmw117. doi:10.1093/fampra/cmw117

11. INVOLVE. *Guidance on Co-Producing a Research Project*.; 2018. https://www.invo.org.uk/wp-content/uploads/2019/04/Copro_Guidance_Feb19.pdf. Accessed March 14, 2018.

12. Deverka PA, Lavallee DC, Desai PJ, et al. Stakeholder participation in comparative effectiveness research: defining a framework for effective engagement. *J Comp Eff Res*. 2012;1(2):181-194. doi:10.2217/cer.12.7

13. Burton H, Adams M, Bunton R, et al. Developing stakeholder involvement for introducing public health genomics into public policy. *Public Health Genomics*. 2009;12(1):11-19. doi:https://dx.doi.org/10.1159/000153426

14. Hansard. Artificial Insemination Of Married Women.

https://api.parliament.uk/historic-hansard/lords/1958/feb/26/artificial-insemination-of-married-women. Published 1958. Accessed March 2, 2020.

15. Mary Barton KW and BPW. Artificial Insemination. 1945;1(4384):40-43.

16. Blyth E. Discovering the “facts of life” following anonymous donor insemination. *Int J Law, Policy Fam*. 2012. doi:10.1093/lawfam/ebs006

17. Davis G. “A tragedy as old as history”: Medical responses to infertility and artificial insemination by donor in 1950s Britain. In: *The Palgrave Handbook of Infertility in History: Approaches, Contexts and Perspectives*. Palgrave Macmillan; 2017:359-381. doi:10.1057/978-1-137-52080-7_19

18. Wellcome Library - Eugenics Society Archive. Advice and Enquiries - Audio interview with Dr Mary Barton. SAEUG/D/3. https://wellcomelibrary.org/item/b1623733x. Accessed August 6, 2020.

19. Stevens B. *Bio-Dad*. Canadian Broadcasting Corporation; 2009. https://web.archive.org/web/20111102185636/http://www.cbc.ca/documentaries/doczone/2009/biodad/.

20. Royal Northern Hospital. Annual report of the Royal Northern Hospital (London, England). https://archive.org/details/b31708900/page/12/mode/2up?q=wiesner. Published 1946. Accessed August 28, 2020.

21. Smith R. British man “fathered 600 children” at own fertility clinic - Telegraph. https://web.archive.org/web/20160810041629/http://www.telegraph.co.uk/news/9193014/British-man-fathered-600-children-at-own-fertility-clinic.html. Accessed February 5, 2020.

22. Human Fertilisation and Embryology Authority. Guide to the Anonymised Register. https://www.hfea.gov.uk/about-us/our-data/guide-to-the-anonymised-register/. Accessed September 17, 2021.

23. Kat Arney, Nunn JS, Middleton A. Hidden family secrets revealed by genetic testing. Genetics Society UK. https://web.archive.org/web/20200203055447/https://geneticsunzipped.com/blog/2020/1/16/family-secrets-revealed-by-genetic-testing. Published 2020.

24. The Canadian Broadcasting Corporation. The World’s Biggest Family. https://www.cbc.ca/cbcdocspov/episodes/the-worlds-biggest-family. Published 2020. Accessed September 1, 2020.

25. Canadian Broadcasting Corporation Radio. How a Toronto woman discovered she has up to 600 half-siblings. https://web.archive.org/save/https://www.cbc.ca/radio/docproject/how-a-toronto-woman-discovered-she-has-up-to-600-half-siblings-1.5727049. Published 2020. Accessed September 30, 2020.

26. Gollancz D. Give me my own history. https://www.theguardian.com/society/2002/may/20/comment.comment. Published 2002. Accessed March 2, 2020.

27. Seawnght J, Gerring J. Case selection techniques in case study research: A menu of qualitative and quantitative options. *Polit Res Q*. 2008.

doi:10.1177/1065912907313077

28. Stake R. *The Art of Case Study Research*.; 1995. https://us.sagepub.com/en-us/nam/the-art-of-case-study-research/book4954. Accessed April 10, 2018.

29. Guest G, MacQueen K, Namey E. *Applied Thematic Analysis*. 2455 Teller Road, Thousand Oaks California 91320 United States : SAGE Publications, Inc.; 2012. doi:10.4135/9781483384436

30. Saldaña J. *The Coding Manual for Qualitative Researchers (2nd Ed.)*. SAGE Publications; 2013. doi:10.1017/CBO9781107415324.004

31. Hyett N, Kenny A, Dickson-Swift V, Dickson-Swift V, Dr. Methodology or method? A critical review of qualitative case study reports. *Int J Qual Stud Health Well-being*. 2014;9:23606. doi:10.3402/qhw.v9.23606

32. Hyett N, Kenny A, Dickson-Swift V. Methodology or method? A critical review of qualitative case study reports. *Int J Qual Stud Health Well-being*. 2014;9(1). doi:10.3402/qhw.v9.23606

33. Yin RK. *Case Study Research: Design and Methods*.; 2009. https://us.sagepub.com/en-us/nam/case-study-research-and-applications/book250150. Accessed April 10, 2020.

34. Houghton C, Casey D, Shaw D, Murphy K. Rigour in qualitative case-study research. *Nurse Res*. 2013;20(4):12-17. doi:10.7748/nr2013.03.20.4.12.e326

35. Gagnier JJ, Kienle G, Altman DG, Moher D, Sox H, Riley D. The CARE guidelines: consensus-based clinical case reporting guideline development. *J Med Case Rep*. 2013;7(1):223. doi:10.1186/1752-1947-7-223

36. Fereday J, Muir-Cochrane E. Demonstrating Rigor Using Thematic Analysis: A Hybrid Approach of Inductive and Deductive Coding and Theme Development. *Int J Qual Methods*. 2006;5(1):80-92. doi:10.1177/160940690600500107

37. Bowen GA. Document Analysis as a Qualitative Research Method. *Qual Res J*. 2009;9(2):27-40. doi:10.3316/qrj0902027

38. Green J, Thorogood N. *Qualitative Methods for Health Research*. SAGE; 2009.

39. Jootun D, McGhee G, Marland GR. Reflexivity: promoting rigour in qualitative research. *Nurs Stand*. 2009;23(23):42-46. doi:10.7748/ns2009.02.23.23.42.c6800

40. Staniszewska S, Brett J, Mockford C, Barber R. The GRIPP checklist: Strengthening the quality of patient and public involvement reporting in research. 2011;4:391-399. doi:10.1017/S0266462311000481

41. Collins M. PiiAF The Public Involvement Impact Assessment Framework Guidance. http://piiaf.org.uk/documents/piiaf-guidance-jan14.pdf. Published 2014. Accessed October 4, 2017.

42. Concannon TW, Meissner P, Grunbaum JA, et al. A new taxonomy for stakeholder engagement in patient-centered outcomes research. *J Gen Intern Med*. 2012;27(8):985-991. doi:10.1007/s11606-012-2037-1

43. INVOLVE. *National Standards for Public Involvement*.; 2018.

http://www.invo.org.uk/posttypepublication/national-standards-for-public-involvement/. Accessed January 29, 2019.

44. Staniszewska S, Brett J, Simera I, et al. GRIPP2 reporting checklists: tools to improve reporting of patient and public involvement in research. 2017. doi:10.1186/s40900-017-0062-2

45. Australian Research Council. *Engagement and Impact Assessment Pilot Report*.; 2018. https://www.arc.gov.au/engagement-and-impact-assessment/ei-pilot-overview. Accessed February 5, 2019.

46. International Collaboration for Participatory Health Research (ICPHR). *Position Paper 1: What Is Participatory Health Research? Version: May 2013*.; 2013. http://www.icphr.org/uploads/2/0/3/9/20399575/ichpr_position_paper_1_defintion_-_version_may_2013.pdf. Accessed June 13, 2017.

47. Jagosh J, Macaulay AC, Pluye P, et al. Uncovering the benefits of participatory research: implications of a realist review for health research and practice. *Milbank Q*. 2012;90(2):311-346. doi:10.1111/j.1468-0009.2012.00665.x

48. Snape D, Kirkham J, Britten N, et al. Exploring perceived barriers, drivers, impacts and the need for evaluation of public involvement in health and social care research: a modified Delphi study. *BMJ Open*. 2014;4(6):e004943. doi:10.1136/bmjopen-2014-004943

49. National Institute for Health Research. *UK Standards for Public Involvement in Research*.; 2019. https://www.invo.org.uk/wp-content/uploads/2019/11/UK-standards-for-public-involvement-v6.pdf.

50. Jack Nunn. Genomics Research and Involving People. La Trobe University Library. https://web.archive.org/web/20200306053056/https://www.youtube.com/watch?v=21TmEfErDcU. Published 2018. Accessed March 6, 2020.

51. Nunn JS. Public involvement activities in 96 global genomics projects. 2018. https://doi.org/10.26181/5b63c24cc1b16.

52. Australian Genomics Health Alliance. What is genomic testing? https://web.archive.org/web/20200306050554/https://www.genomicsinfo.org.au/wp-content/uploads/2019/02/What-is-genomic-testing-v9.pdf. Published 2019. Accessed March 6, 2020.

53. Genome BC. What is Genomics? https://web.archive.org/web/20191221110313/https://www.youtube.com/watch?v=mmgIClg0Y1k. Published 2010. Accessed March 6, 2020.

54. Nunn JS, Tiller J, Fransquet PD, Lacaze P. Public Involvement in Global Genomics Research: A Scoping Review. *Front Public Heal*. 2019;7:79. doi:10.3389/FPUBH.2019.00079

55. U.S National Library of Medicine. What does it mean if a disorder seems to run in my family? https://web.archive.org/web/20180529060700/https://ghr.nlm.nih.gov/primer/inheritance/runsinfamily. Published 2018. Accessed March 6, 2020.

56. Genomics England. Data in the 100,000 Genomes Project (version 1.0 02/09/15) .

https://web.archive.org/web/20200306055926/https://m.youtube.com/watch?v=nneWFaJ6Hfc. Published 2015. Accessed March 6, 2020.

57. Centre for Genetics Education. Ethical issues in human genetics and genomics. https://web.archive.org/web/20200306060134/https://www.genetics.edu.au/publications-and-resources/facts-sheets/fact-sheet-19-ethical-issues-in-human-genetics-and-genomics. Published 2018. Accessed March 6, 2020.

58. Cancer Research UK. Genes, DNA and cancer . https://web.archive.org/web/20200306060347/https://www.cancerresearchuk.org/about-cancer/what-is-cancer/genes-dna-and-cancer. Published 2017. Accessed March 6, 2020.

59. American Society of Clinical Oncology. How to Share Genetic Test Results With Family . https://web.archive.org/web/20200306060636/https://www.cancer.net/blog/2017-03/how-share-genetic-test-results-with-family. Published 2017. Accessed March 6, 2020.

60. Genomics England. How do you sequence a human genome? https://web.archive.org/web/20200306060635/https://www.genomicsengland.co.uk/wp-content/uploads/2015/10/Seq-infographic-FNL-061015-01-01HI-RES-01.jpg. Published 2015. Accessed March 6, 2020.

61. Sally Davies et al. *Annual Report of the Chief Medical Officer 2016 - "Generation Genome."*; 2017. https://www.gov.uk/government/uploads/system/uploads/attachment_data/file/624628/CMO_annual_report_generation_genome.pdf.

62. Cancer Australia. Priority-driven Collaborative Cancer Research Scheme. https://canceraustralia.gov.au/research-data/research/priority-driven-research. Published 2018. Accessed February 2, 2018.

63. Kelty C, Panofsky A. Disentangling public participation in science and biomedicine. *Genome Med*. 2014;6(1):8. doi:10.1186/gm525

64. Brett J, Staniszewska S, Mockford C, et al. Mapping the impact of patient and public involvement on health and social care research: a systematic review. *Health Expect*. 2014;17(5):637-650. doi:10.1111/j.1369-7625.2012.00795.x

65. Crowe S, Fenton M, Hall M, Cowan K, Chalmers I. Patients', clinicians' and the research communities' priorities for treatment research: there is an important mismatch. *Res Involv Engagem*. 2015;1(1):2. doi:10.1186/s40900-015-0003-x

66. World Health Organisation. Declaration of Alma-Ata. http://www.who.int/publications/almaata_declaration_en.pdf?ua=1. Published 1978. Accessed June 25, 2018.

67. Nunn JS, Gwynne K, Crawshaw M, Lacaze P. Involving people in genomics research. October 2019. doi:10.26181/5DA78C5CED9D5

68. Shakespeare W. The Tempest. https://www.bl.uk/works/the-tempest. Accessed February 8, 2020.

69. Nunn JS, Sulovski M, Tiller J, Holloway B, Ayton D, Lacaze P. Involving elderly

research participants in the co-design of a future multi-generational cohort study. *Res Involv Engagem*. 2021;7(1):23. doi:10.1186/s40900-021-00271-4

70. Black E. *IBM and the Holocaust: The Strategic Alliance Between Nazi Germany and America's Most Powerful Corporation*. Dialog Press; 2018. http://www.dialogpress.com/books/ibm-and-the-holocaust/1.

71. Wiggins J, Middleton A. *Getting the Message across : Communication with Diverse Populations in Clinical Genetics*.; 2013.

72. Middleton A, Hewison J, Mueller RF. Attitudes of deaf adults toward genetic testing for hereditary deafness. *Am J Hum Genet*. 1998;63(4):1175-1180. doi:10.1086/302060

73. Seethamraju R. Effectiveness of Using Online Discussion Forum for Case Study Analysis. *Educ Res Int*. 2014;2014:1-10. doi:10.1155/2014/589860

74. National Research Ethics Service. Patient and public involvement in research and research ethics committee review. https://web.archive.org/web/20160509095054/http://www.invo.org.uk/wp-content/uploads/2011/12/INVOLVENRESfinalStatement310309.pdf. Published 2009.

75. Pandya-Wood R, Barron DS, Elliott J. A framework for public involvement at the design stage of NHS health and social care research: time to develop ethically conscious standards. *Res Involv Engagem*. 2017;3(1):6. doi:10.1186/s40900-017-0058-y

76. Kemmis S, Nixon R, McTaggart R. *The Action Research Planner: Doing Critical Participatory Action Research.*; 2014. doi:10.1007/978-981-4560-67-2
