# Supplementary material for: Co-designing genomics research with a large group of donor-conceived siblings
Source: Res Involv Engagem. 2021 Dec 16;7:89. doi: 10.1186/s40900-021-00325-7 (PMC8674833; doi:10.1186/s40900-021-00325-7)
Supplement: Supplementary file 2 — Additional file 2. STARDIT report. [file 40900_2021_325_MOESM2_ESM.pdf]

Standardised Data on Initiatives (STARDIT) report – Alpha Version

About this report

This report uses the Standardised Data on Initiatives Alpha version (STARDIT)[1]. The 'living' STARDIT Beta version[2] report which relates to this project can be found in the references[3].

STARDIT Report Alpha Version

**Identifying information**	
Initiative name	Co-designing genomics research with donor -conceived siblings
Geographic location or scope	Australia
Date range (planned start and end dates of initiative)	2017-2020
Purpose of the initiative	Participatory action research to involve members of a sibling group in online discussions about how they would like to be involved in future research.
Organisations or other initiatives involved (list all if multi-centre)	1. School of Psychology and Public Health, La Trobe University
Funding sources	School of Psychology and Public Health, La Trobe University
Clinical trial registration details (if applicable)	N/A
Ethics approval (if applicable)	La Trobe University
Other relevant information (free text)	This report describes involving members of a sibling group in online discussions about how they would like to be involved in future research.
At which stage of the research project has this report been written? (Select from: 1. **Before** the intervention or initiative– this report is prospective or describes planned activity 2. **Ongoing** – the intervention or initiative is still taking place 3. **After** the research project or initiative has occurred	**After** the participatory action research occurred.
Methods of the initiative (what is planned to be done, or is being reported as done)	The research process was co-designed using a participatory action research method to involve members of a sibling group in the co-design of online discussions to explore future genomic research with members of the group.
**Report authorship**	
Name	1: Jack Nunn<br>2: Marilyn Crawshaw

This STARDIT report (Alpha version) contains additional data relevant to the case study 'Co-designing genomics research with a large group of donor-conceived siblings' [https://doi.org/10.21203/rs.3.rs-100595/v1].

	3: Paul Lacaze<br>4: Shirley Brailey<br>5: Barbara Nunn<br>6: Adrianne Smith<br>7: Barry Stevens
Public domain profiles, institutional pages	1: https://scholars.latrobe.edu.au/display/j2nunn<br>2: https://www.york.ac.uk/spsw/staff/emeritus-and-honorary/marilyn-crawshaw/<br>3: https://www.monash.edu/medicine/sphpm/about/staff/academic/lacaze<br>4: N/A<br>5: N/A<br>6: N/A<br>7: https://www.wikidata.org/wiki/Q4864782
Open Researcher and Contributor ID (orcid.org)	1: https://orcid.org/0000-0003-0316-3254<br>2: https://orcid.org/0000-0002-2870-0506<br>3: https://orcid.org/0000-0002-0902-6798
Tasks in report completion	1: Main report author<br>2: Checked report data<br>3: Checked report data<br>4: Checked report data and contributed additional data<br>5: Checked report data and contributed additional data<br>6: Checked report data and contributed additional data<br>7: Checked report data and contributed additional data
Other information	Other people were involved in writing this report and contributing data but did not want to be named.
Key contact at initiative for confirming report content (include institutional email address)	Jack Nunn, PhD researcher, School of Psychology and Public Health, La Trobe University, jack.nunn@latrobe.edu
**Involvement**	
Who was involved or how would you label groupings of those involved	Group 1: Academic research investigators (Jack Nunn, Marilyn Crawshaw and Paul Lacaze)<br>Group 2: Members of the sibling group who gave feedback during the co-design stage (including but not limited to Becky Gardiner, David Gollancz and Michael Bywater)<br>Group 3: Members of the sibling group who participated in the research and gave feedback as part of the co-design process, the manuscript checking stage or contributed data to the STARDIT report (including but not limited to Shirley Brailey, Barbara Nunn, Adrianne Smith and Barry Stevens)
How many people were in each grouping label?	Group 1: 3<br>Group 2: 5<br>Group 3: 18

Specific tasks of this person or group (list as many as possible) – *including any information about why certain people were included or excluded in certain tasks*	Group 1: Involved in co-designing every stage of the process, analysing data<br>Group 2: Members of the sibling group were involved in refining wording of participant information, sharing views and advice about the process, proof-reading documents, providing feedback on surveys, analysing data, informing planning, and providing feedback on planned online discussions.<br>Group 3: Participants were also involved in checking the content of Genetics Society UK podcast[4], with the recording shared with all participants before dissemination to ask them to check the content was accurate and acceptable.<br>Group 2 and 3: Participants were sent the article and additional files to check the analysis and content and were invited to be authors of the STARDIT report.
How were these people involved (what methods were used)	Group 1: Face-to-face meetings, video calls, email communication, shared online documents, teleconferences.<br>Group 2: video calls, email communication, shared online documents, teleconferences.
Facilitators of involvement (what do you expect will help these people get involved – or what helped them get involved)	Giving people time to read resources. Clear communication about the intention of involving people.<br><br>One participant noted that being 'highly educated' was an enabler for involvement and that having a 'bit of time on their hands' was also an enabler [P4]. Being 'respectful' when involving 'those affected by genomic research' will facilitate research as the 'more brains applied to research, the more likely answer to puzzles will be found' [P11]. Similarly, another participant stated 'the more diverse the debate, the more dilute the effect of irrational preconception and ethical incompetence should become' [P8]. One participant stated that whatever model was chosen, it should be 'as flexible as possible' [P5].<br><br>Four participants reported specific things about the way this study was conducted that enabled their involvement. One participant said the entire process was 'assiduous' and that the 'intent of this project' was 'obviously thoughtful and interesting'[P9]. One participant said the 'system seemed to work well' [P7]. Another added that being used to online platforms like Loomio, or having previous experience of similar platforms and 'used to' that way of communicating might facilitate involvement using that communication mode. One participant suggested an alternative discussion format where the participants discussed a thread for 2 days and then had a 3-day break before coming to another thread [P7].<br><br>The Facilitator (MC) noted that regular contact with the study team and timely support was essential and they 'could not have done it without this'.
Barriers of involvement (what do you expect will inhibit these people from getting involved – or what inhibited them from getting involved). Are there any known equity issues which may contribute?	Face-to-face meetings were difficult to organise. The study team was located in both Australia and the UK, so face-to-face meetings were not possible. Unclear communication about intentions and purpose of the involvement contributed to confusion (explaining how involvement is distinct from participation was challenging). Ensuring those involved had enough time to give feedback was also a challenge.

	Barriers to involvement in research identified by participants included public fear and 'hysteria' caused by a lack of understanding, which may 'hamper' research, involvement, and general public support for research [P5]. Synchronous discussion was highlighted as another barrier if participants 'are across time zones' [P4]. One participant mentioned that they felt that their emotional response to some issues made it difficult to get involved in some ways [P6]. Being required to watch lengthy videos was identified as a barrier by one participant. One-to-one interviews were mentioned as being 'quite straight-jacketed with circumscribed questions' compared to more open online discussions [P4]. They also stated 'I don't think that a group discussion would work as there are too many voices and some would be drowned out' [P4]<br><br>Four participants reported specific things about the way this study was conducted that were barriers to their involvement. A discussion about boundaries revealed that some participants felt 'avoiding topics which might trigger emotions which are stressful or unpleasant' could be viewed as 'restrictive, even censorious' [P7]. The pace of the discussions was mentioned as moving 'too quickly' with another adding 'more time' was needed and study team should 'reconsider the pace of the research' [P7] [P4] [P5]. Updates from the discussion were sent to participants according to their preferences, and one stated they 'lost track of emails' and were sometimes unsure if they were 'responding to the right part' [P6]. Two participants stated the 'platform presented technical difficulties' [P4] and that it was 'complicated' [P5]. One participant stated the 'premise and the purpose of the study could be clearer' and that the various discussion threads were 'difficult to untangle sometimes' [P7]. They also mentioned it was 'hard to be able to guarantee to do this every day for a period' and that not doing so meant they 'got lost' [P7]. Another participant added that 'it's a difficult subject to discuss in a vacuum, without real life examples' [P4]. One participant expressed 'trepidation' at sharing views about research and compared the feeling to getting an answer wrong in an 'exam' [P6].<br><br>The Facilitator (MC) stated that they felt more time was required in the co-design process.
What was the outcome or output of the involvement of these people? What changed as a result of involving people?	Improved participant information resources, improved wording that was culturally appropriate (using terminology preferred by the sibling group to describe biological relations), improved online discussion, improved learning resources for participants, improved co-design process.
At which stage of the initiative were these people involved? (select from list of pre-defined stages or allow 'other')	Group 1: All stages<br>Group 2: Co-design, evaluation and dissemination
What was the estimated financial cost for involving people. How much time did it take. Were there any costs that cannot be measured financially?	$0 AUD – people volunteered their time. The total number of hours volunteered (excluding participation and the contributions of co-investigator MC) is estimated to be 25.

What worked well, what could have been improved? Was anything learned from the process of involving these people?	Involving potential participants in co-defining language used to describe the sibling group helped ensure that language was acceptable and appropriate.<br><br>The co-design process took longer than expected owing to ethical 'grey areas' with no clear instruction on whether ethics approval was required to involve people in co-design. As a result an ethics application was made and subsequent feedback from the co-design process was integrated using modifications to the ethics application.<br><br>The entire study team agreed that limitations in the ethics process affected the extent of how the sibling group could be involved in the study. Internationally, confusion still surrounds what ethical approval is required before involving potential participants in co-designing research. On the advice of the La Trobe University Human Research Ethics Committee, the study team did not approach potential participants about co-designing the study until after ethics approval had been granted, with feedback from participants being incorporated by a number of subsequent modifications to the original ethics application. As a result of the complex process of modifications, the timeline for feedback was shorter than the study team had anticipated, although the process did provide useful feedback.<br><br>Ambiguous policies for the ethical involvement of people in co-designing research can hamper the degree of control potential participants have in research and further clarity from ethics committees will enhance power sharing at this crucial stage of research.
**Mapping financial or other 'interests'**	
Describe any financial relationship or other interest this person has to this project	One investigator (Jack Nunn) is biologically related to participants from the sibling group, with one being his mother and all being half-aunts or uncles.
Describe any conflicting or competing interests	N/A
**Data**	
Who is the data from this intervention shared with?	It will be published open access in peer reviewed journals with identifying information removed in order to anonymise it as much as possible.
How is it stored and hosted?	It will be shared on a public domain repository.
Who is analysing the data?	Group 1: The study team described above<br>Group 2: participants were invited to review the analysis and give feedback to ensure they felt it reflected their experience of the process
What methods will be used to analyse the data (including a link to any relevant code and information about validity)	We used case study methodology to describe our experience involving participants in the co-design of the proposed study. We collected and analysed both qualitative and quantitative data during the involvement activities.<br><br>We analysed data from online surveys and online discussions with participants. In addition, data from the study team communications was included, such as meeting notes, emails, reflexive diary entries and

	survey responses of study investigators. Coding and thematic analysis of qualitative data was carried out by two authors independently and checked by other authors.
How is information about this data disseminated?	1. It will be published in an open access journal<br>2. It will be shared with participants of the research and also other members of the sibling group who have joined it since the study commenced<br>3. Learning from this process has been presented at conferences, and will be shared on social media and through other channels. Preliminary learning was shared in a UK Genetics Society podcast[4].
Who 'owns' the data or claims any kind of 'intellectual property' (include relevant licensing information)	Confidential data collected as part of the study is stored according to laws and the data access plan approved by La Trobe University.<br><br>The authors maintain 'ownership' of the data in the paper and is shared under the Creative Commons license used by the publishing journal.
Who controls access to the data	The study team, La Trobe University and participants will be involved in any future data access decisions.
How is/will the data be 'Findable, Accessible, Interoperable, Reusable' according to the FAIR criteria?	Data will be shared in the public domain and licensed under a Creative Commons license.
**Impacts and outcomes**	
What new knowledge has been generated? (if appropriate, include effect size, relevant statistics and level or evidence)	1. Involving participants in co-designing the research process resulted in a number of changes to the study design, including improving language used in recruitment and learning resources<br>2. The process of involving people can be viewed as a learning experience for both the participants involved and study team members. The process changed participants' views about who should be involved, which can be viewed as an impact of 'transformative learning'.
What was learned	Involving people in online discussions about involvement in research changes people's views about who should be involved in research, including participants 'widening' their views about who should be involved in research to include more people.
Knowledge translation	1. Knowledge from this process will inform the design of a future genomic research<br>2. Learning from this process can inform future involvement activities<br>3. Learning from this co-design process can inform future ways of involving people in genomic research including co-designing self-governed biobanks.<br>4. Learning from this process was shared in a Genetics Society UK podcast[4], with the recording shared with all participants before dissemination to ensure the content was accurate and acceptable.

Outcomes	1. **Co-design changed study design.** Feedback from participants resulted in changes to the study design including improving language used in recruitment, improving the online discussions and learning resources.<br>2. **The process improved participants understanding about genomics and research**. Participants had an improved understanding of genomics. While participants showed a good-baseline level understanding of genomics, three reported their understanding about genomics and research increased as a result of participating in the study. However, some participants demonstrated a lack of self-confidence in their understanding, in spite of demonstrating a good understanding of the principles of genomic research, citing relevant peer-reviewed literature in discussions and discussing the nuances of ethical oversight.<br>3. **Participants' improved understanding about genomics and research helped them make informed decisions about invitations to join genomics research studies**, which were shared with members of the sibling group after the process by researchers unconnected with this study.<br>4. **Learning from this process informed subsequent discussions in the sibling group about participation in research**, including a proposed self-managed biobank. Method for future research co-design established. By co-creating methods of involving participants in proposed future genomics research, this process has demonstrated a practical and well-evaluated method of involving potential participants in co-designing research. Participants stated that the methods used in this process could be helpful when co-designing future stages of proposed genomic research with the sibling group.<br>5. **Participants reported finding the learning resources useful** (including infographics and videos). One participant commented they had learned from the visual summary of the review of public involvement in genomic research stating 'I didn't realise there were so many research projects involving global genomics projects but it is so good to read that public involvement is becoming more and more important' [P5]<br>6. **Participants changed views and perspectives about genomics research as a result of participating.** Four out of the six participants who completed the follow up survey noted their views and perspectives changed as a result of participating. One participant stated 'I now realise how fast the field of genomics is changing and there are all kinds of implications especially in the field of precision medicine' [P5]. One participant also indicated that involvement in research might help people make sense of their personal experience and added joining an online discussion 'let me think beyond my emotion' [P6]. Another stated 'involving people in genomic research is crucial' as it has 'unknown consequences and needs as wider discussion as possible'[P4]. A number of participants had views about 'leaving research to the qualified' challenged by other participants [P9], with follow-up survey data suggesting that those challenged changed their views about who should be involved, towards widening. The changed

	views of the participants involved can be viewed as an impact of 'transformative learning'.<br>7. **Participants asked to stay involved in the research.** All participants who completed the follow-up survey requested to stay involved in the research process, including in analysing data and being co-authors on the paper<br>8. **Participants enjoyed the online discussions**. Participants stated the experience of participating was 'interesting' and they 'enjoyed thinking about the questions posed and reading the responses of others' and the 'perceptive comments' of the Facilitator [P7] [P4]. Another participant added that it 'worked well' as a way of involving people [P7].
How has or how will this be measured?	Future STARDIT reports
Who is involved in measuring this?	The study team and participants

References

1. Nunn J, Shafee T, Chang S, et al. Standardised Data on Initiatives - STARDIT: Alpha Version. 2019. doi:10.31219/osf.io/5q47h

2. Nunn JS, Shafee T. Standardised Data on Initiatives – STARDIT: Beta Version. doi:10.31219/osf.io/w5xj6

3. Nunn JS, Crawshaw M, Lacaze P, et al. *Co-Designing Genomics Research with Donor-Conceived Siblings (STARDIT Beta Version Report)*. https://www.wikidata.org/wiki/Q108618394. Accessed September 7, 2021.

4. Kat Arney, Nunn JS, Middleton A. Hidden family secrets revealed by genetic testing. Genetics Society UK. https://web.archive.org/web/20200203055447/https://geneticsunzipped.com/blog/2020/1/16/family-secrets-revealed-by-genetic-testing. Published 2020.
