# Supplementary material for: Co-designing genomics research with a large group of donor-conceived siblings
Source: Res Involv Engagem. 2021 Dec 16;7:89. doi: 10.1186/s40900-021-00325-7 (PMC8674833; doi:10.1186/s40900-021-00325-7)
Supplement: Supplementary file 3 — Additional file 3. GRIPP2 report. [file 40900_2021_325_MOESM3_ESM.pdf]

GRIPP2 report for ‘Involving people affected by a rare condition in shaping future genomic research’

This report has been completed using the ‘GRIPP2 reporting checklists: tools to improve reporting of patient and public involvement in research’ available at https://doi.org/10.1136/bmj.j3453.

GRIPP2 short form

Section and topic	Category description	Data
1: Aim	Report the aim of PPI in the study	Participatory action research to involve members of a sibling group in online discussions about how they would like to be involved in future research.
2: Methods	Provide a clear description of the methods used for PPI in the study	The research process was co-designed using a participatory action research method to involve people from the sibling group in the co-design of online discussions to explore future genomic research with members of the group. Participants were also involved in checking the final version of the paper.
3: Study results	Outcomes—Report the results of PPI in the study, including both positive and negative outcomes	Improved participant information resources, improved wording that was culturally appropriate (using terminology preferred by the sibling group to describe biological relations), improved online discussion, improved learning resources for participants, improved co-design process.<br><br>Enablers of involvement: Four participants reported specific things about the way this study was conducted that enabled their involvement. One participant said the entire process was ‘assiduous’ and that the ‘intent of this project’ was ‘obviously thoughtful and interesting’[P9]. One participant said the ‘system seemed to work well’ [P7]. Another added that being used to online platforms like Loomio, or having previous experience of similar platforms and ‘used to’ that way of communicating might facilitate involvement using that communication mode. One participant

Section and topic	Category description	Data
		suggested an alternative discussion format where the participants discussed a thread for 2 days and then had a 3 day break before coming to another thread [P7].<br><br>Barriers of involvement: Four participants reported specific things about the way this study was conducted that were barriers to their involvement. A discussion about boundaries revealed that some participants felt 'avoiding topics which might trigger emotions which are stressful or unpleasant' could be viewed as 'restrictive, even censorious' [P7]. The pace of the discussions was mentioned as moving 'too quickly' with another adding 'more time' was needed and study team should 'reconsider the pace of the research' [P7] [P4] [P5]. Updates from the discussion were sent to participants according to their preferences, and one stated they 'lost track of emails' and were sometimes unsure if they were 'responding to the right part' [P6]. Two participants stated the 'platform presented technical difficulties' [P4] and that it was 'complicated' [P5]. One participant stated the 'premise and the purpose of the study could be clearer' and that the various discussion threads were 'difficult to untangle sometimes' [P7]. They also mentioned it was 'hard to be able to guarantee to do this every day for a period' and that not doing so meant they 'got lost' [P7]. Another participant added that 'it's a difficult subject to discuss in a vacuum, without real life examples' [P4]. One participant expressed 'trepidation' at sharing views about research and compared the feeling to getting an answer wrong in an 'exam' [P6].<br><br>The Facilitator (MC) stated that they felt more time was required in the co-design process.
4: Discussion and conclusions	Outcomes—Comment on the extent to which PPI influenced the study overall. Describe positive and negative effects	Involvement improved participant information resources, improved wording that was culturally appropriate (using terminology preferred by the group to describe themselves), improved online discussion, improved learning resources for participants, improved co-design process.

Section and topic	Category description	Data
		Involving potential participants in co-defining language used to describe the group of people affected helped ensure that language was acceptable and appropriate.<br><br>Involving participants in co-designing the research process resulted in a number of changes to the study design, including improving language used in recruitment and learning resources.<br><br>The process of involving people can be viewed as a learning experience for both the participants involved and study team members. The process changed participants' views about who should be involved, which can be viewed as an impact of 'transformative learning'.
5: Reflections/critical perspective	Comment critically on the study, reflecting on the things that went well and those that did not, so others can learn from this experience	The co-design process took longer than expected owing to ethical 'grey areas' with no clear instruction on whether ethics approval was required to involve people in co-design. As a result an ethics application was made and subsequent feedback from the co-design process was integrated using modifications to the ethics application.<br><br>Involving potential participants in co-defining language used to describe the group of people affected helped ensure that language was acceptable and appropriate.<br><br>Involving participants in co-designing the research process resulted in a number of changes to the study design, including improving language used in recruitment and learning resources.<br><br>The process of involving people can be viewed as a learning experience for both the participants involved and study team members. The process changed participants' views about who should be involved, which can be viewed as an impact of 'transformative learning'.

Section and topic	Category description	Data
		Involving people in online discussions about involvement in research changes people’s views about who should be involved in research, including participants ‘widening’ their views about who should be involved in research to include more people.
